# The meristem-associated endosymbiont *Methylorubrum extorquens* DSM13060 reprograms development and stress responses of pine seedlings

**DOI:** 10.1093/treephys/tpab102

**Published:** 2021-07-29

**Authors:** Janne J Koskimäki, Johanna Pohjanen, Jouni Kvist, Thomas Fester, Claus Härtig, Olga Podolich, Silvia Fluch, Jaanika Edesi, Hely Häggman, Anna Maria Pirttilä

**Affiliations:** Ecology and Genetics Research Unit, University of Oulu, Paavo Havaksentie J1, FI-90014 Oulu, Finland; Ecology and Genetics Research Unit, University of Oulu, Paavo Havaksentie J1, FI-90014 Oulu, Finland; Stem Cells and Metabolism Research Program, Faculty of Medicine, University of Helsinki, Haartmaninkatu 8, FI-00014 Helsinki, Finland; Department of Environmental Microbiology, Helmholtz Centre for Environmental Research – UFZ, Permoserstr. 15, 04318 Leipzig, Germany; Department of Environmental Microbiology, Helmholtz Centre for Environmental Research – UFZ, Permoserstr. 15, 04318 Leipzig, Germany; Institute of Molecular Biology and Genetics of NASU, Acad. Zabolotnoho str., 150 03680 Kyiv, Ukraine; Weiden am See, Burgenland 7121, Austria; Ecology and Genetics Research Unit, University of Oulu, Paavo Havaksentie J1, FI-90014 Oulu, Finland; Production Systems, Tree Breeding, Natural Resources Institute Finland LUKE, FI-57200 Savonlinna, Finland; Ecology and Genetics Research Unit, University of Oulu, Paavo Havaksentie J1, FI-90014 Oulu, Finland; Ecology and Genetics Research Unit, University of Oulu, Paavo Havaksentie J1, FI-90014 Oulu, Finland

**Keywords:** intracellular, metabolism, plant–microbe interactions, symbiosis, transcription network

## Abstract

Microbes living in plant tissues—endophytes—are mainly studied in crop plants where they typically colonize the root apoplast. Trees—a large carbon source with a high capacity for photosynthesis—provide a variety of niches for endophytic colonization. We have earlier identified a new type of plant–endophyte interaction in buds of adult Scots pine, where *Methylorubrum* species live inside the meristematic cells. The endosymbiont *Methylorubrum extorquens* DSM13060 significantly increases needle and root growth of pine seedlings without producing plant hormones, but by aggregating around host nuclei. Here, we studied gene expression and metabolites of the pine host induced by *M. extorquens* DSM13060 infection. Malic acid was produced by pine to potentially boost *M. extorquens* colonization and interaction. Based on gene expression, the endosymbiont activated the auxin- and ethylene (ET)-associated hormonal pathways through induction of *CUL1* and *HYL1*, and suppressed salicylic and abscisic acid signaling of pine. Infection by the endosymbiont had an effect on pine meristem and leaf development through activation of *GLP1-7* and *ALE2*, and suppressed flowering, root hair and lateral root formation by downregulation of *AGL8*, *plantacyanin*, *GASA7*, *COW1* and *RALFL34*. Despite of systemic infection of pine seedlings by the endosymbiont, the pine genes *CUL1*, *ETR2, ERF3*, *HYL*, *GLP1-7* and *CYP71* were highly expressed in the shoot apical meristem, rarely in needles and not in stem or root tissues. Low expression of *MERI5, CLH2*, *EULS3* and high quantities of ononitol suggest that endosymbiont promotes viability and protects pine seedlings against abiotic stress. Our results indicate that the endosymbiont positively affects host development and stress tolerance through mechanisms previously unknown for endophytic bacteria, manipulation of plant hormone signaling pathways, downregulation of senescence and cell death-associated genes and induction of ononitol biosynthesis.

## Introduction

As plants have a sessile lifestyle, they are forced to interact with the microbes they encounter at the spot, whether a pathogen, a mutualist or a harmless commensalist. Plants may therefore have developed a higher tolerance for microbes to live within their tissues than animals. Some of the plant-associated microbes settle for the available nutrients on the plant surfaces, whereas others penetrate the surface and grow inside plant tissues. Endophytes, bacteria or fungi that live in plant tissues without eliciting visible symptoms, are found in every plant species in high diversity (Petrini 1986, Rosenblueth and Martínez-Romero 2006). Importance of endophytes in plant in defense, stress tolerance and promotion of plant growth has been reported (Rosenblueth and Martínez-Romero 2006, Hardoim et al. 2015). The majority of bacterial endophytes was first discovered in the intercellular spaces of root tissues, where their nitrogen fixation was studied (Rosenblueth and Martínez-Romero 2006). Today, the significance of endophytic nitrogen fixation is considered low, but still, most endophyte studies are performed on root-associated endophytes of crop plants (Hardoim et al. 2015). However, the long-living trees, which possess a high capacity for photosynthesis and comprise a large carbon source, are natural targets for microbes, either as resources, or partners (Friesen et al. 2011). The long age of trees combined with immobility require a long-lasting resistance against pathogens (Carroll 1988, Arnold et al. 2003), as well as the capacity to respond to changes in environmental conditions (Shen and Fulthorpe 2015).

We have identified a novel plant–endophyte interaction in the bud tissues of adult Scots pine (*Pinus sylvestris* L.) trees, where bacteria of the genera *Methylorubrum* (earlier *Methylobacterium*) and *Pseudomonas*, and a yeast, *Rhodotorula minuta* live inside the meristematic cells of the shoot tips (Pirttilä et al. 2000, 2003). By in situ hybridization, we have shown that the *Methylorubrum* spp. are the most common endosymbionts throughout the year, being present in buds of every tree examined (Pirttilä et al. 2000, 2005). Bacteria in the genus *Methylorubrum* are generally found as epiphytes on many different plant species, such as soybean, common bean and rice (Holland 1997, Madhaiyan et al. 2004). On leaf surfaces, the communities of *Methylorubrum* spp. are supported by continuous emissions of methanol originating from plant cell-wall biosynthesis (Fall 1996), as these bacteria are methylotrophic, i.e., they can utilize methanol as the sole source of carbon and energy (Patt et al. 1976).

Endosymbiotic *Methylorubrum* spp. are found in pine shoot tips prior to bud elongation and development but not during bud growth or dormancy (Pirttilä et al. 2005). The endosymbiont *M. extorquens* DSM13060, which has been isolated from Scots pine buds, colonizes pine seedlings through mechanisms similar to those of stem-colonizing rhizobia (Koskimäki et al. 2021). The genome of *M. extorquens* DSM13060 hosts a number of *Nod-like genes*, which supports the similarities found in colonization mechanisms with the rhizobial relatives (Koskimäki et al. 2015). The bacterium utilizes the plant-emitted methanol as an energy source for actively entering the host through cylindrical sheath and epidermis in roots, as well as stomatal apertures in shoots, and forms infection pocket-like structures upon entry. By forming infection thread-like structures, the endosymbiont invades deeper tissues, endoderm and vascular system, through which it systemically colonizes the in vitro-grown Scots pine seedlings (Koskimäki et al. 2021).

The inoculation of pine seedlings with *M. extorquens* DSM13060 significantly increases growth of needles and roots, at levels comparable to growth induction by mycorrhizal fungi (Pohjanen et al. 2014). Whereas epiphytic *Methylorubrum* strains typically produce plant hormones (Ivanova et al. 2001, Koenig et al. 2002), those are not found in *M. extorquens* DSM13060 (Pirttilä et al. 2004, Koskimäki et al. 2015). The bacterium aggregates around the nuclei of living host cells, and the genome encodes nucleomodulins, eukaryotic transcription factors, which may interfere with host transcription and metabolism (Koskimäki et al. 2015). However, detailed responses of the host, Scots pine, to the infection by *M. extorquens* DSM13060 are not known at the molecular level. Here we studied changes in gene expression and metabolic responses of Scots pine seedlings infected by *M. extorquens* DSM13060, to elucidate the specific mechanisms of the host–microbe interaction.

## Materials and methods

### Plant material

Seeds of *P. sylvestris* elite line (K884) were incubated in 55 °C for 72 h and transferred to sterile water overnight. The seeds were subsequently surface sterilized with 3% calcium hypochlorite (w/v) for 15 min and rinsed thoroughly three times with sterile water. The seeds were planted into glass jars containing sterile vermiculite and water and grown at 22 ± 1 °C, 16-h photoperiod and irradiance of 75 μmol m^−2^ s^−1^ for 5 days. After germination, each seedling was inoculated by pipetting 2.5 × 10^6^ CFU of *M. extorquens* DSM13060 diluted in sterile water, either the wild-type or a strain carrying a chromosomal green fluorescent protein (GFP) reporter tag (Pohjanen et al. 2014). Growing was continued for 90 days. Upon sample collection, all seedlings were immediately frozen in liquid nitrogen and stored at −80 °C until gene expression and metabolomics analyses.

### RNA isolation and cDNA synthesis

For the RNA extraction, 10 pine seedlings were pooled as one sample. RNA was isolated from the seedlings according to the first part of the protocol described by Jaakola et al. (2001). After LiCl_2_ precipitation, the RNA pellet was treated with RNase-free DNase set (Qiagen, Hilden, Germany) for 15 min at 25 °C to digest any contaminating DNA. Reactions containing the total RNA were subsequently purified using RNeasy Mini Kit (Qiagen, Hilden, Germany). The RNA concentrations were quantified using NanoDrop® ND-1000 spectrofotometer (NanoDrop Technologies, Wilmington, DE, USA). RNA quality was assessed by agarose gel electrophoresis and using Bioanalyzer 2100 (Agilent Technologies, Santa Clara, CA, USA) with RNA Nano 6000 chips. Each total RNA sample (40 μg/reaction) was reverse transcribed to cDNA using Superscript III reverse transcriptase (Thermo Fisher Scientific, Vilnius, Lithuania) with oligo-dT primers incorporated with dye-specific (Cy3 or Cy5) capture sequences according to the protocol of 3DNA Array 50 Expression Array Detection Kit (Genisphere, Hatfield, PA, USA). We used custom cDNA microarray chips (PICME Pine 7K, Austrian Institute of Technology, Seibersdorf) in a total of six hybridization reactions to minimize technical variability and dye bias. The cDNAs printed on the PICME_Pine arrays were produced by INRA. The arrays consist cDNAs isolated as putative drought-responsive genes of *Pinus pinaster*, and thus represent an enriched subset of stress-responsive genes of pine (Dubos and Plomion 2003).

### Transcriptional profiling

Microarray chips were prewashed in 2 × SSC, 0.2% SDS at 65 °C for 10 min, followed by a wash in 0.2 × SSC at room temperature for 2 min and rinse in deionized water for 1 min at room temperature. The hybridization and washing procedures were performed according to the protocol of 3DNA Array 50 Expression Array Detection Kit (Genisphere, Hatfield, PA, USA) recommended for the PCR product (cDNA) arrays, where the unlabeled first-strand cDNAs are first hybridized to the microarray chip, and after a washing step, the microarray chips are incubated with fluorescently pre-labeled 3DNA dendrimers (Cy3 and Cy5) containing the capture sequence for the cDNAs. The cDNA hybridization mix consisted of 10 μl of dye-specific cDNAs from *M. extorquens*-inoculated and untreated control pine seedling, 2 μl of locked nucleic acids dT blocker, 13 μl of nuclease free water and 25 μl of 2 × formamide-based hybridization buffer. The cDNA hybridization was carried out in a dark humidified hybridization chamber (Corning Inc., Corning, NY, USA) at 45 °C for 16 h. Hybridized microarray slides were washed in 2 × SSC, 0.2% SDS for 15 min at 65 °C, 2 × SSC for 10 min at room temperature, and in 0.2 × SSC for 10 min at room temperature with gentle agitation. Fluorescent labelling of the cDNAs by 3DNA hybridization was carried out in a total volume of 50 μl using a formamide-based buffer with 3 h incubation at 48 °C in a dark humidified hybridization chamber according to the manufacturer’s protocol recommended for the PCR product (cDNA) arrays. Hybridized microarray slides were washed as previously and immediately spin-dried in a Falcon-tube by centrifugation at 250 × *g* for 2 min. Slide scanning was performed with a ScanArray Gx microarray scanner (Perkin Elmer, Waltham, MA, USA) with 90% laser power at 5-micron resolution. The photomultiplier tube (PMT) values were adjusted for each scanning to minimize the background noise. Fluorescent images were analyzed with ScanArray Express software (Perkin Elmer, Waltham, MA, USA), and low-quality spots were flagged and removed during the statistical analysis.

### Primer design

We used *Pinus* unigene library (DFGI Gene Index [PGI] ver. 9, released on 26 March 2011) and the Sitka spruce (*Picea sitchensis*) cDNA library (Ralph et al. 2008) to construct gene contigs of *P. sylvestris* L. sequences that correspond to the *P. pinaster* cDNAs on the PICME microarrays. *Pinus sylvestris* L. -specific PCR primers were designed against the conserved regions of the contigs, and the gene sequences were amplified with Phusion high-fidelity DNA polymerase (Life Technologies, Carlsbad, CA, USA) using the cDNA as a template. The PCR products were cloned into pJET 1.2/blunt cloning vector (Thermo Fisher Scientific, Waltham, MA, USA) and sequenced using BigDye terminator cycle sequencing kit (v3.1, Applied Biosystems, Foster City, CA, USA) on a 3730 DNA Analyzer (Applied Biosystems, Foster City, CA, USA). Based on the obtained *P. sylvestris* L. sequences, 10 gene-specific real-time quantitative PCR (RT-qPCR) primer pairs were designed for the genes that showed significant differential expression in the microarray experiment. Both PCR and RT-qPCR primers (Table 1) were designed using Primer3 software (version 3.0.0; Untergasser et al. 2012).

**Table 1:** RT-qPCR primers used in this study. Corresponding *Pinus taeda* gene object IDs and *Arabidopisis thaliana* locus identifiers are provided for each gene

Gene name	*P. taeda* ID	*A. thaliana* locus	Primer sequences	Primer names
Actin 7 (*ACT7*)	PITA_000001835	AT5G09810	CAGCGGGTATCCATGAGACT AACCTCCGATCCAAACACTG	*Ps*ACT7-F *Ps*ACT7-R
Chlorophyllase 2 (*CLH2*)	PITA_000058463	AT5G43860	TCAGAGTTCGGACACATGGA ACTGCGGAGGTTCGAGTATG	*Ps*CLH2-F *Ps*CLH2-R
Cullin 1 (*CUL1*)	PITA_000025623	AT4G02570	GGGCATCTTTGTGGAGATTG CAGCAACTTTGGTTCACTGGT	*Ps*CUL1-F *Ps*CUL1-R
Cytochrome P450 (*CYP71*)	PITA_000050150	AT5G07990	TGGATTGGCTCGATTTGC GTCAAGCACAACAGCCTTGAT	*Ps*CYP75B1-F *Ps*CYP75B1-R
Glyceraldehyde-3-phosphate dehydrogenase C2 (*GAPC2*)	PITA_000019953	AT1G13440	GCGCAGAGTATGTGGTTGAA AAAGGGGCCAAGCAGTTAGT	*Ps*GAPC2-F *Ps*GAPC2-R
Germin-Like Protein 1 member 7 (*GLP1-7*)	PITA_000010539	AT3G05950	TGAAGGCGAGTCCAGTCC ACAGATAGCCCTTGCGTTTG	*Ps*GLP1-F *Ps*GLP1-R
Ethylene-responsive transcription factor 3 (*ERF3*)	PITA_000025077	AT5G25190	AGCGGCAAGGATGATGTG CAAGTGAGGGACTGATTGCTG	*Ps*ESE3-F *Ps*ESE3-R
Ethylene Response 2 (*ETR2*)	PITA_000020141	AT3G23150	CCCTCACTATGCGAAGGTTTT CACCCTTGGAGTTAGGCTCA	*Ps*ETR1-F *Ps*ETR1-R
Hyponactic Leaves 1 (*HYL1*)	PITA_000066063	AT1G09700	CTGTCGAGATTGCAGGGATT CAGAGGGTTGGTCAGATGCT	*Ps*HYL1-F *Ps*HYL1-R
Thaumatin/Osmotin like protein (Pathogenesis-Related protein 5, *PR-5*)	*P. pinaster* HIT: sp_v3.0_unigene33770	AT4G11650	ACGTACTGGATGCTCCTTCG TCGGAGGGAGTTAGGGAGAT	*Ps*PR5-F *Ps*PR5-R

### RT-qPCR

Five micrograms of total RNA of *M. extorquens*-inoculated and untreated control pine seedlings (both 7 and 90 days post-inoculation, DPI) was reverse transcribed to cDNA using SuperScript III First-Strand Synthesis System (Thermo Fisher Scientific, Vilnius, Lithuania) as described earlier. The real-time qPCR was performed in white 96-well PCR plates (Roche Diagnostics, Mannheim, Germany) using a LightCycler 480 instrument (Roche Diagnostics, Mannheim, Germany). Each PCR reaction (14 μl) consisted of 2× SYBR green master mix (7 μl; Roche Diagnostics, Mannheim, Germany), 4-μM forward and reverse primers (1.75 μl each) and cDNA sample (3.5 μl; 1/50 dilution). The PCR program consisted an initial denaturation at 95 °C for 10 min, followed by 45 cycles at 95 °C for 10 s (ramp rate 4.4 °C/s), 60 °C for 20 s (ramp rate 2.2 °C/s) and 72 °C for 10 s (ramp rate 4.4 °C/s). The melting curve was measured at 95 °C for 0.5 s (ramp rate 4.4 °C/s), 57 °C for 15 s (ramp rate 2.2 °C/s) and 98 °C for 0 s (ramp rate 0.11 °C/s). All samples and controls were amplified in five biological replicates with technical repeats. Relative expression levels were normalized to *GAPDH* and *ACTIN7* reference genes, and the results were calculated with LightCycler 480 software (release 1.5.1.62 SP3) using calibrator-normalized relative quantification with efficiency correction (E-method, Tellmann 2006). The calculated normalized fold changes with both reference genes were comparable and, therefore, only the results obtained with *GAPDH* are presented in the main text.

### In situ hybridization

The inoculated and control seedlings were surface sterilized in 70% ethanol for 1 min, and in 4% calcium hypochlorite for 15 min. After rinsing in sterile water, the seedlings were dissected, the needles and roots were cut into 5-mm pieces and apical meristems were cut longitudinally. The tissues were then fixed in 0.1 M NaH_2_PO_4_–Na_2_HPO_4_ (pH 7.4), 2% paraformaldehyde and 2.5% glutaraldehyde at 4 °C overnight. The tissues were dehydrated, cleared through an ethanol–*t*-butanol series, and embedded in paraffin (Merck, Whitehouse Station, NJ, USA). In situ hybridization was performed as described by Pirttilä et al. (2000). Briefly, the paraffin-embedded tissues were cut to 6-μm thick sections, baked on silane-coated slides and paraffin was removed in xylene. Slides were air-dried, fixed and treated with sodium borohydride and proteinase K, as described previously (Pirttilä et al. 2000). Each cDNA fragment was amplified from reverse-transcribed RNA of Scots pine and cloned into the vector pGEM-T Easy (Promega, Madison, WI, USA). Digoxigenin-labeled sense and antisense RNA probes were prepared by in vitro transcription using the DIG RNA (SP6/T7) Labeling Kit (Roche Diagnostics, Mannheim, Germany).

Hybridization was performed aseptically one to three times per sample. The slides were hybridized in a hybridization buffer containing 3× SET (450-mM NaCl, 60-mM Tris–HCl pH 7.5 and 3-mM EDTA), Denhardt’s solution (0.02% Ficoll, Sigma-Aldrich, 0.02% polyvinyl pyrrolidone, Sigma-Aldrich, 0.02% bovine serum albumin, Sigma-Aldrich), 0.02% tRNA (Sigma-Aldrich), 0.02% polyadenylic acid (Sigma-Aldrich, Saint Louis, MO, USA), 10% dextran sulfate (Merck, Darmstadt, Germany), 50-mM dithiothreitol (Merck), 50% formamide and the probe (0.05 ng/μl). Slides containing the hybridization mixture were placed in a hybridization chamber saturated with 3× SET, and incubated at 42 °C overnight. After the hybridization, slides were washed with 2× SET at RT for 15 min and with 0.1× SET at 55 °C for 15 min. Detection was performed with the DIG Nucleic Acid Detection kit (Roche Diagnostics, Mannheim, Germany), after which the slides were rinsed with 70% ethanol, air-dried and viewed under bright-field microscope.

### Confocal laser scanning microscopy

Pine seedlings inoculated with *M. extorquens* DSM13060 carrying the GFP tag were cut to 3-mm pieces and fixed under vacuum in 4% paraformaldehyde (w/v), 0.1% glutaraldehyde (v/v), 20% glycerol (v/v) and 0.1 M sodium phosphate buffer (pH 7.4) for 4 h at +4 °C. Using a cryomicrotome (Reichert-Jung 2800 Frigocut with 2040 microtome, Leica Microsystems, Wetzlar, Germany), 20–30-μm sections were cut and mounted on microscopy slides with 0.1 M sodium phosphate buffer (pH 7.4) containing 10% glycerol (v/v). The sections of pine seedlings (roots and shoots) were viewed with a confocal laser scanning microscope (LSM 5 Pascal, Carl Zeiss, Jena, Germany) using Plan-Neofluor 40×/1.3 and Plan-Apochromat 63×/1.4 oil objectives. We used the wavelength of 488 nm by argon ion laser to excite the GFP reporter and the 505–530-nm band-pass filter for detecting the emission. A 650 Long-pass filter was used to collect background autofluorescence. The resulting images were analyzed and merged using Zeiss ZEN software (ver. ZEN 2012 blue edition, Carl Zeiss Microscopy GmbH, Jena, Germany).

### Metabolite profiling

Metabolite profiling was based on the procedure described by Sanchez et al. (2008). The plant material, 57–64 mg of needles, stems and roots either from *M. extorquens*-inoculated seedlings, or untreated controls, was frozen in liquid nitrogen in 8–16 replicates. The frozen plant material was homogenized in a Retsch-ball mill for 3 min at 30 s^−1^ and re-suspended in 300-μl methanol at −20  °C. After the addition of 30-μl ribitol (0.2 mg/ml dissolved in methanol), samples were incubated in a shaker for 15 min at +70 °C. Subsequently, 200-μl chloroform was added, samples were shaken for 5 min at +37 °C, mixed with 400 μl of water and vortexed. Phase separation was achieved by centrifugation (5 min, 14,000 rpm). Two 10-μl aliquots from the upper phase were finally dried in vacuum overnight at room temperature. The dried material was stored at −80 °C and derivatized as described by Desbrosses et al. (2005). The samples were suspended in 80-μl methoxamin hydrochloride (20 mg/ml in pyridine), incubated for 90 min at 30 °C. Subsequently, 80 μl of *N*-methyl-*N*-(trimethylsilyl)-trifluoroacetamide was added, and samples were incubated for 30 min at 37 °C. Finally, 16 μl of a standard mix containing C_10_, C_12_, C_15_, C_18_, C_19_, C_22_, C_28_, C_32_, C_36_  *n*-alkanes each at 0.22 mg/ml was added. Gas chromatography/mass spectrometry was done using an Agilent GC 6890 (Agilent Technologies, Waldbronn, Germany) equipped with a Rtx-5Sil MS capillary column (30 m × 0.25 mm inner diameter, 0.25-μm film thickness and 5-m integrated guard column; Restek GmbH, Bad Homburg, Germany) and an MSD 5973. One microliter of each sample was injected in split mode (10:1) at a temperature of 230 °C. Helium was used as carrier gas with constant flow at 1 ml/min (adjusted by retention time locking). The temperature program was 1 min at 70 °C, 1 °C/min to 76 °C and finally 6 °C/min to 350 °C, held for 1 min. The transfer line to the mass spectrometer was set to 300 °C. Baseline correction of gas chromatography–mass spectrometry data, chromatographic deconvolution and quantification of compounds was done using MassProfiler Professional 12.6 (Agilent Technologies, Waldbronn, Germany) and MetaboAnalyst 2.0 (Xia et al. 2012).

### Statistical analyses

#### Transcriptional profiling

Differential expression analysis was carried out with Limma (Ritchie et al. 2015). The raw intensities within each array were normalized with Loess-method (with background subtraction), and quantile was normalized among arrays. Differential expression was analyzed with linear regression (lmFit), using contrast between the inoculated and control samples. The treatment was a fixed effect, and technical variation among replicated probes was a random effect in the model (using duplicateCorrelation-function). The variance was smoothed with eBayes. The expression changes were false discover rate corrected with BH-method (Benjamini and Hochberg 1995). The heatmap for differentially expressed genes (adjusted *P* value < 0.01) was done using ComplexHeatmap (v2.4.3; Gu et al. 2016). All differential analyses were carried out with the R software (R Core Team 2019). Functional annotation and gene ontology (GO) classification was done using the Blast2Go pipeline implemented in OmicsBox (BioBam). In short, cDNAs were first blasted (BlastN) against *P. pinaster* SustainPine v3.0. unigenes to find the corresponding full-length sequences. The obtained gene sequences were then compared (BlastX) against the annotated protein sequences of *Pinus taeda* (v. 1.01) and *Arabidopsis thaliana* (TAIR10) genomes, and the functions of each known protein was retrieved from Enzyme Commission (EC), InterPro (IPR) and UniProt (https://www.uniprot.org). The gene set enrichment analysis (GSEA) for gene ontology terms was done with clusterProfiler (v3.16.1; Yu et al. 2012), using the custom GO term associations. Genes were sorted by fold change and analyzed for enrichment using 100 permutations without a *P* value cutoff.

#### RT-qPCR

After examination of normal distribution of data using the Shapiro–Wilk’s test, and equality of variances using the Levene’s test, pairwise comparisons of expression of each tested gene in the inoculated and control samples was done using either *t*-test or Wilcoxon test. In case the data were normally distributed, the *t*-test was used, and Wilcoxon signed-rank test was used for non-normally distributed datasets in the program R (R Core Team 2019).

#### Metabolite profiling

Statistical analysis was performed using MassProfiler, and in pairwise comparisons, using MetaboAnalyst 2.0 (Xia et al. 2012). In MassProfiler, the missing data were replaced by ‘1’ and data were logarithmized and filtered using the size of the relative standard deviation. Entities were eliminated when no treatment could be found with a respective signal in at least 50% of measurements. Multivariate ordination analysis of metabolite levels from the whole dataset was performed using principal component analysis (PCA). In MetaboAnalyst 2.0, the missing data were replaced by half of the minimum positive values in the original data. The data were filtered using the size of the relative standard deviation, normalized row-wise to constant sums and logarithmized. Analyses by MassProfiler and MetaboAnalyst 2.0 revealed that 49 samples (9 samples in the case of control needles and 8 samples in the case of all other tissue and treatment groups) could be used for statistical analysis. Data processing using MassProfiler resulted in 136 peaks aligned in all chromatograms. Multivariate ordination analysis in pairwise comparisons was then done using partial least square-discriminant analysis (PLS-DA; Naes and Mevik 2001, Wold et al. 2001). Significance of the separation of treatment groups in such pairwise comparisons was tested by permutational multivariate analysis of variance (PERMANOVA, 999 permutations) using the function ‘adonis’ of the R-package ‘vegan’ (Oksanen et al. 2012). Significance of differences in the levels of individual metabolites was evaluated by combining a *t*-test (*P* < 0.1) and a fold change analysis (fold change > 2 or < 0.5; volcano-plot) in MetaboAnalyst 2.0. Retention time indices (RI) were calculated from the added *n*-alkanes. Metabolites were identified by comparison of the locked retention time index and the mass spectrum with the Golm metabolome database (Kopka et al. 2005, Schauer et al. 2005) using AMDIS (Stein 1999).

## Results

**Figure 1.**
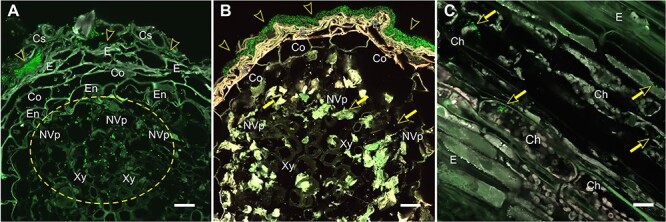
Colonization of Scots pine (*Pinus sylvestris* L.) roots and stem by *Methylorubrum extorquens* DSM13060 at 90 DPI. (A) Bacterial colonies reside in the cylindrical sheath and epidermis (arrowheads), accumulating in the non-vascular parenchyma and in the xylem vessels (circled) of root. (B) Biofilm-like bacterial growth covering the root surface (arrowheads) and individual bacterial cells in the non-vascular parenchyma. (C) Bacteria inside chlorenchymal cells (arrows) of stem. Note: The bacterial cells carrying a fluorescent GFP are visualized in bright green. Microscopic sections: Cross (A, B); longitudinal (C). Ch = chlorenchyma, Co = cortex, Cs = cylindrical sheath, E = epiderm, En = endoderm, NVp = non-vascular parenchyma, Xy = xylem. Scale bar—20 μm.

**Figure 2.**
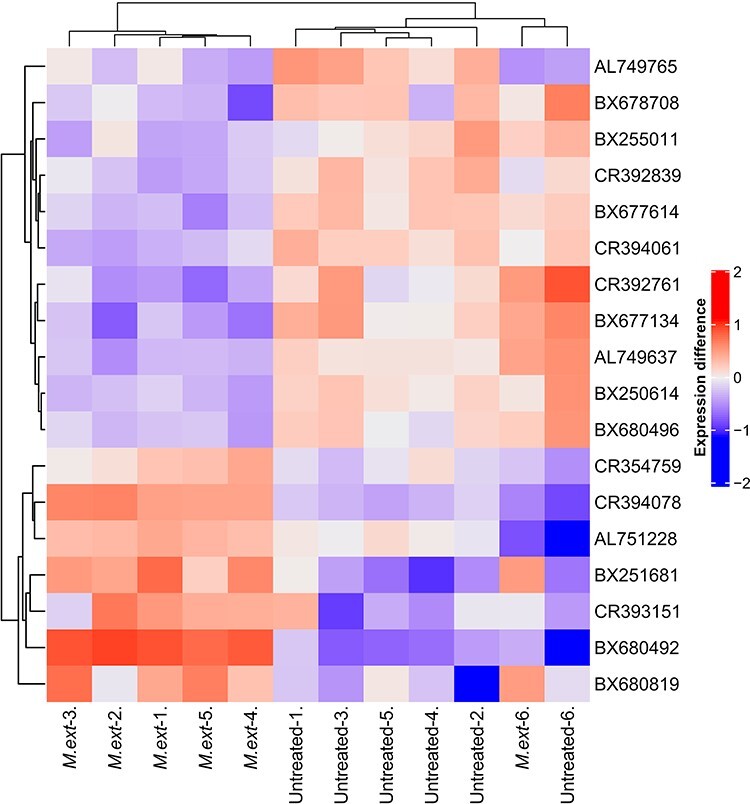
Heatmap of differentially expressed genes (adj. *P* < 0.01) between six replicates of *Methylorubrum extorquens* DSM13060-inoculated (‘*M ext*’) and control (‘untreated’) Scots pine seedlings. The heatmap is showing differences in expression compared with gene averages. Normalized intesites were scaled with log1p, and the gene averages were subtracted from individual expressions.

**Figure 3.**
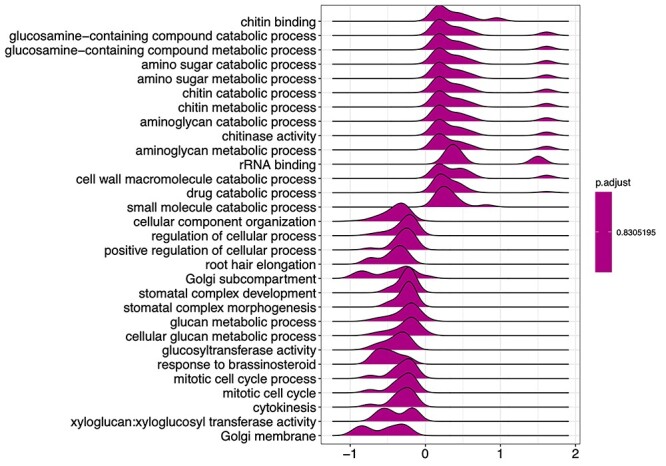
Gene set enrichment analysis for gene ontology terms of up- or downregulated genes in Scots pine due to *Methylorubrum extorquens* DSM13060 infection. Genes were sorted by fold change and analyzed for enrichment using 100 permutations without a *P* value cutoff.

We selected the time point of 90 DPI for our analysis based on our earlier observations that at this point, *M. extorquens* DSM13060 has systemically colonized the pine seedlings, being found throughout tissues of roots and shoots (Figure 1). The *M. extorquens*-infected seedlings and control samples had different expression profiles, where technical variability among replicates within treatment was low, evaluated by heatmap analysis (Figure 2). Infection of pine seedlings by *M. extorquens* DSM13060 significantly (*P* < 0.05) increased (>0.47 log fold change, logFC) expression of 68 known genes, and decreased (<−0.47 logFC) expression of 66 known genes (see Table S1 available as Supplementary data at *Tree Physiology* Online). The upregulated genes were roughly involved in chitin binding and chitinase activity, glucosamine-containing compound and amino sugar metabolism, aminoglycan metabolism, rRNA binding, cell wall and small molecule catabolic processes, whereas the main categories of downregulated genes included cellular component organization, regulation of cellular process, root hair elongation, stomatal complex development, glucan metabolism, response to brassinosteroid, mitotic cell cycle and xyloglucan:xyloglucosyl transferase activity (Figure 3).

**Figure 4.**
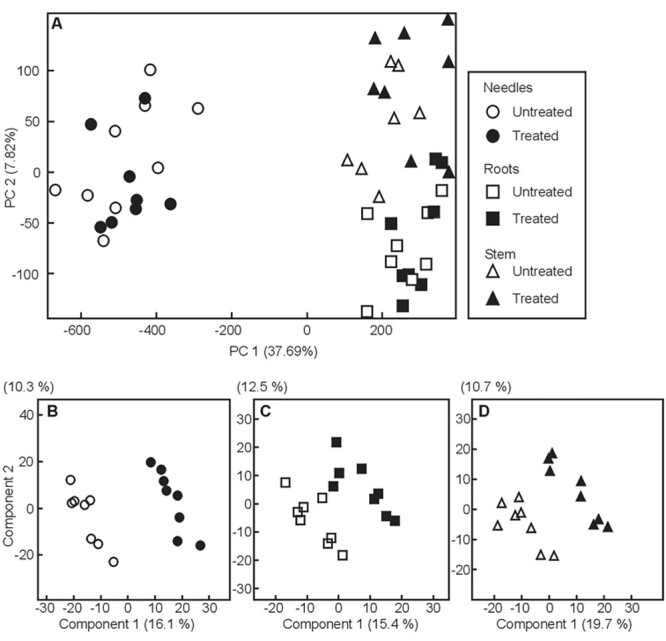
Multivariate analysis of the complete data set using PCA (A) and pairwise comparisons of 90 days old *Methylorubrum extorquens*-inoculated (black symbols) and untreated (white symbols) pine seedlings for needles (B, circles), roots (C, rectangles) and stems (D, triangles) using partial least square-discriminant analysis (PLS-DA).

A PCA on the metabolome dataset at the 90 DPI timepoint revealed a strong impact of the plant organ (needles *versus* stem, or roots), separating (37.7% of data variance) photosynthetic from non-photosynthetic organs (Figure 4A). Data processing by PLS-DA resulted in 862, 709 and 716 peaks for the organs studied, needles, roots and stems, respectively, separating the treatments (Figure 4B–D). According to PERMANOVA analysis, this separation was significant in the case of needles (*P* < 0.001).

### Hormonal signaling

**Figure 5.**
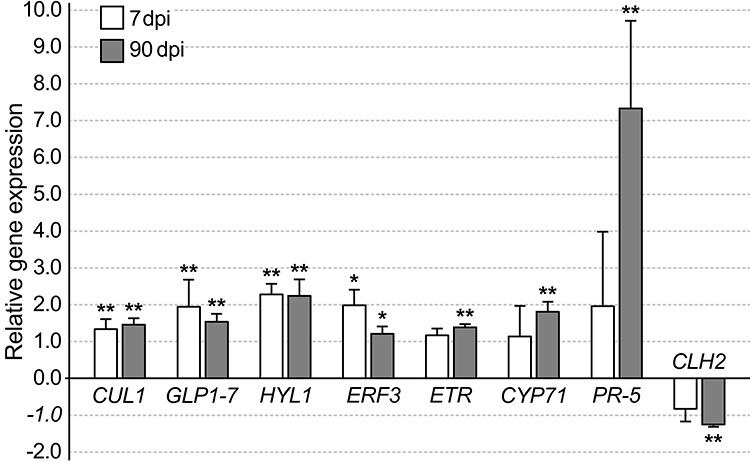
A bar graph showing normalized relative gene expression of *Methylorubrum extorquens* DSM13060-inoculated pine seedlings 7 and 90 DPI analyzed by real-time quantitative PCR. Fold changes are relative to *GAPDH*, *, *P* < 0.05, **, *P* < 0.01 indicate statistical significance between comparisons of treatment and control. *CUL1* = Cullin1, *CYP71* = cytochrome P450, *ERF3* = ethylene-responsive transcription factor 3, *HYL1* = Hyponactic leaves 1, *PR-5* = pathogenesis-related protein 5.

**Figure 6.**
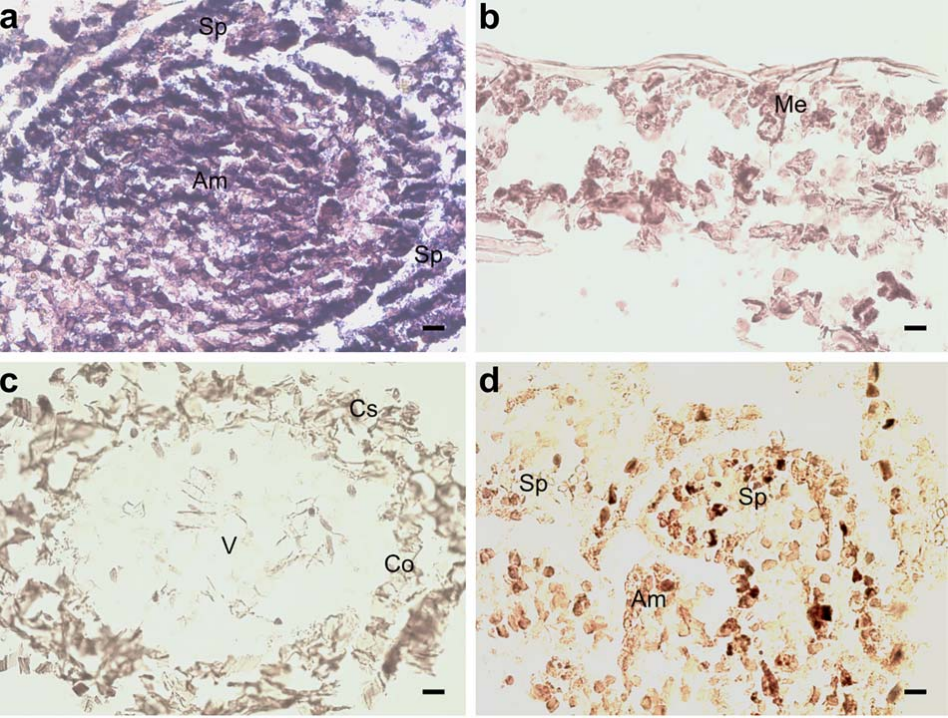
In situ hybridization of pine seedlings inoculated with *Methylorubrum extorquens* and controls 90 DPI. (A) Shoot tip meristem, (B) needle and (C) root tissue of an inoculated seedling hybridized with a probe specific for *CUL1*. (D) Shoot tip meristem of control seedling hybridized with a probe specific for *CUL1*. Am = apical meristem, Sp = scale primordium, Me = mesophyll, V = vascular tissue, Co = cortex and Cs = cylindrical sheath. Scale bar—20 μm.

Indicative of auxin signaling, the gene *CR354759* similar with *A. thaliana* auxin-response factor 2, ARF2 (*AT5G62000*) had increased expression at 0.47 logFC (*P* < 0.01 and adj. *P* < 0.01), and *BX254429* similar to Cullin 1 (CUL1) (*AT4G02570*) had increased expression at 0.97 logFC (*P* < 0.05) in the *M. extorquens*-inoculated seedlings than in controls according to the transcriptional profile analysis (see Table S1 available as Supplementary data at *Tree Physiology* Online). When analyzed by RT-qPCR, *CUL1* had 1.3–1.5 times higher expression in the inoculated seedlings 7 and 90 DPI (Figure 5). Based on in situ hybridization, *CUL1* had the highest expression in the shoot tip meristems (Figure 6A) and a strong expression in needles (Figure 6B), but none in root tissues (Figure 6C). The gene had weak expression in meristems of the control seedlings (Figure 6D). In contrast, *BX680071* similar with the myb-related protein MYB44 (*AT5G67300*) that activates the transcription of the auxin-responsive gene IAA19, was downregulated by 0.60 logFC (*P* < 0.01) in the inoculated seedlings compared with controls (see Table S1 available as Supplementary data at *Tree Physiology* Online).

**Figure 7.**
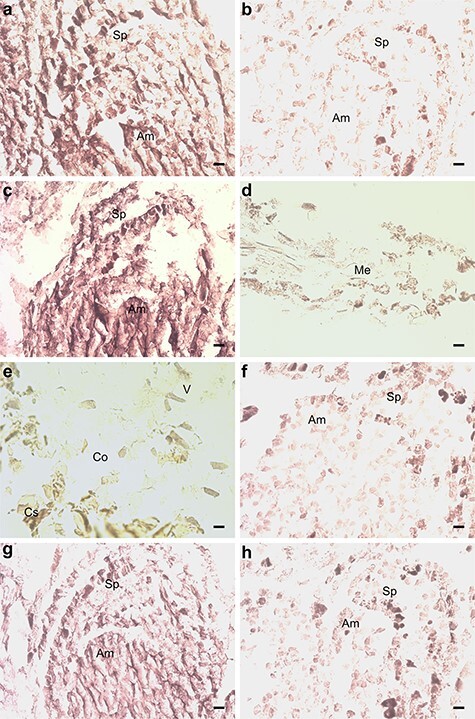
In situ hybridization of pine seedlings inoculated with *Methylorubrum extorquens* and controls 90 DPI. (A) Shoot tip meristem of an inoculated and (B) a control seedling, hybridized with a probe specific for the *ETR2.* (C) Shoot tip meristem, (D) needle and (E) root tissue of an inoculated seedling, hybridized with a probe specific for the *ERF3*. (F) Shoot tip meristem of a control seedling hybridized with a probe specific for the *ERF3*. (G) Shoot tip meristem of an inoculated and (H) a control seedling, hybridized with a probe specific for *HYL1*. Am = apical meristem, Sp = scale primordium, Me = mesophyll, V = vascular tissue, Co = cortex and Cs = cylindrical sheath. Scale bar—20 μm.

Several genes associated with ethylene (ET) signaling were up- or downregulated in the *M. extorquens*-inoculated seedlings compared with controls. *BX253641* similar with ethylene responsive factor 3 (ERF3) (*AT5G25190*) had increased expression by 0.64 logFC (*P* < 0.05), *BX679686* similar with ethylene response factor 4 (ERF4) (*AT3G15210*) had increased expression by 1.69 logFC (*P* < 0.05), and *BX682473* similar with the ethylene binding receptor ethylene response 2 (ETR2) (*AT3G23150*) was upregulated by 0.69 logFC (*P* < 0.01; see Table S1 available as Supplementary data at *Tree Physiology* Online). When analyzed by RT-qPCR, *ETR2* had 1.2–1.4 times higher expression 7 and 90 DPI with *M. extorquens* DSM13060 (Figure 5). By in situ hybridization analysis, the gene had high expression in the shoot tip meristems of the infected seedlings (Figure 7A), compared with low expression in the controls (Figure 7B). *ERF3* had 2.0 times higher expression 7 DPI, which descended to 1.2-fold expression in 90 days (Figure 5). This gene was strongly expressed in the shoot tip meristems of infected pine seedlings (Figure 7C), whereas no expression was observed in the needles or roots (Figure 7D and E), and a weak expression was present in the shoot tips of controls (Figure 7F). There was also one downregulated gene, *CR394371* similar with ethylene responsive transcription factor ERF010/DEAR2 (*AT5G67190*), by −0.66 logFC (*P* < 0.05), in the inoculated seedlings (see Table S1 available as Supplementary data at *Tree Physiology* Online).

Associated with gibberellin signaling, *BX250160* similar with GA-stimulated Arabidopsis 6 protein (GASA6) (*AT1G74670*), had 0.56 logFC (*P* < 0.01) expression in the inoculated seedlings compared with controls (see Table S1 available as Supplementary data at *Tree Physiology* Online). The *M. extorquens*-infected seedlings had −0.87 logFC expression (*P* < 0.01 and adj. *P* < 0.05) of the gene *CR392755* similar with a glycine-rich protein 23 (ATGRP23) (*AT2G32690*), and −0.85 logFC expression (*P* < 0.01 and adj. *P* < 0.05) of *BX254845*, similar with nucleotide-sugar transporter family protein (ROCK1) (*AT5G65000*), compared with controls (see Table S1 available as Supplementary data at *Tree Physiology* Online).

### Epigenetics, micro-RNA and RNA interference

The gene *AL749800* similar to Hyponastic Leaves 1 (HYL1) (*AT1G09700*), associated with micro-RNA binding, had increased expression by 2.24 logFC (*P* < 0.01) in inoculated seedlings than in controls (see Table S1 available as Supplementary data at *Tree Physiology* Online), and the RT-qPCR analysis confirmed a 2.2–2.3-fold higher expression 7 and 90 DPI (Figure 5). *HYL1* expression was localized by in situ hybridization to the tissues of shoot meristem both in inoculated and control seedlings (Figure 7G and H). *BX678370*, a gene similar to ORRM2 (*AT5G54580*), which is responsible for RNA editing in mitochondria, had 2.77 logFC expression (*P* < 0.05), and the gene *BX682105* similar with AT3G05060, a protein with a role in snoRNA binding, had 1.50 logFC expression (*P* < 0.01) in inoculated seedlings than in controls (see Table S1 available as Supplementary data at *Tree Physiology* Online). In contrast, *BX253783* similar with TEX1 (*AT5G56130*) involved in the trans-acting small interfering RNA (ta-siRNA) pathway, had −2.05 logFC expression (*P* < 0.05), and *BX680222* similar with FK506 binding protein 53 (FKBP53) (*AT4G25340*), a histone chaperone that represses 18S rDNA expression, had −1.23 logFC expression (*P* < 0.01 and adj. *P* < 0.05) in *M. extorquens*-inoculated seedlings compared with controls (see Table S1 available as Supplementary data at *Tree Physiology* Online).

### Cell wall biosynthesis

The gene *BX250930*, which is similar with xyloglucan endotransglucosylase/hydrolase 9 XTH9 (*AT4G03210*) that participates in cell wall construction of growing tissues, had decreased expression by −1.01 logFC (*P* < 0.05), and the gene *BX679601* similar with HXXXD-type acyl-transferase family protein involved in suberin biosynthesis (*AT5G41040*) had −0.76 logFC expression (*P* < 0.05) in *M. extorquens*-inoculated seedlings compared with controls (see Table S1 available as Supplementary data at *Tree Physiology* Online). Furthermore, *CR394328* similar with FASCICLIN-like arabinogalactan-protein 12 (FLA12; *AT2G20520*) had −0.49 logFC expression (*P* < 0.01), and *CR354742* similar to CGR2 protein (*AT3G49720*), which has a role in plant growth and esterification of homogalacturonan pectins in the Golgi apparatus, had −0.65 logFC expression (*P* < 0.01 and adj. *P* < 0.05) in the inoculated seedlings compared with controls (see Table S1 available as Supplementary data at *Tree Physiology* Online).

### Intra- and intercellular trafficking

Two genes, *BX680146* and *BX677372*, similar with the plasmodesmata-located protein PDLP8 (*AT3G60720*) of Arabidopsis, had increased expression by 0.60 logFC (*P* < 0.05 for both), and *BX255736* similar with RAB GTPase homolog H1E (RABH1e) (*AT5G10260*) had increased expression by 1.02 logFC (*P* < 0.01) in the *M. extorquens*-inoculated seedlings compared with controls (see Table S1 available as Supplementary data at *Tree Physiology* Online). Correspondingly, *BX678356* that was similar with the cell-to-cell mobile mRNA element ZCF37 (*AT1G59590*), had expression decreased by 0.62 logFC (*P* < 0.05) in the inoculated seedlings (see Table S1 available as Supplementary data at *Tree Physiology* Online).

### Lipid metabolism and transport

The gene *BX254421* similar with the long-chain alcohol oxidase FAO3 (*AT3G23410*) had 1.47 logFC expression (*P* < 0.01), and *BX253270* that resembles the *O*-fucosyltransferase family protein (*AT3G49210*) had 0.87 logFC expression (*P* < 0.05) in the *M. extorquens*-inoculated seedlings compared with controls (see Table S1 available as Supplementary data at *Tree Physiology* Online). There were four genes, *BX250678, CR393500, BX677658* and *BX680131*, similar with protease inhibitor/lipid transfer proteins (LTPs) (*AT2G37870, AT5G59310* and *AT3G53980*), which had expression reduced by 0.56–0.62 logFC (*P* < 0.01 for all) in the inoculated seedlings (see Table S1 available as Supplementary data at *Tree Physiology* Online). Furthermore, *BX255011* similar with bifunctional inhibitor/lipid-transfer protein (*AT3G53980*) had −0.52 logFC expression (*P* < 0.01 and adj. *P* < 0.01), and *BX252727* similar with GDSL esterase/lipase (*AT4G01130*) had −0.52 logFC expression (*P* < 0.05) in the inoculated seedlings compared with the controls (see Table S1 available as Supplementary data at *Tree Physiology* Online).

### Embryogenesis and meristem development

**Figure 8.**
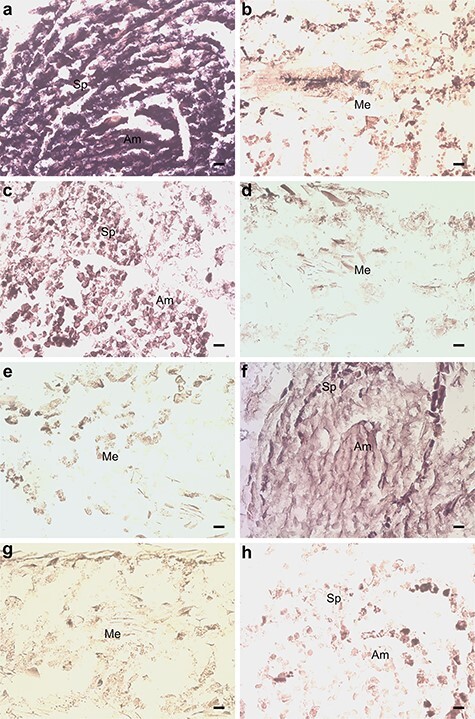
In situ hybridization of pine seedlings inoculated with *Methylorubrum extorquens* and controls 90 DPI. (A) Shoot tip meristem and (B) needle tissue of an inoculated seedling, hybridized with a probe specific for *GLP1-7*. (C) Shoot tip meristem and (D) needle tissue of a control seedling, hybridized with a probe specific for *GLP1-7*. (E) Needle tissue of an inoculated seedling, hybridized with a sense probe specific for *GLP1-7*. (F) Shoot tip meristem and (G) needle tissue of an inoculated seedling, hybridized with a probe specific for *CYP71*. (H) Shoot tip meristem of a control seedling, hybridized with a probe specific for *CYP71*. Am = apical meristem, Sp = scale primordium, Me = mesophyll, V = vascular tissue, Co = cortex, Cs = cylindrical sheath. Scale bar—20 μm.

Overall, expression of a large group of genes associated with development was altered in *P. sylvestris* seedlings due to *M. extorquens* infection. Two genes, *CR393282* and *BX251681*, similar with a Germin-like protein subfamily 1 member 7 (GLP1-7) (*AT3G05950*) and Germin-like protein 2 (GLP2a) (*AT5G39190*), had increased expression by 2.16 (*P* < 0.01 and adj. *P* < 0.05) and 1.48 logFC (*P* < 0.01 and adj. *P* < 0.01) in the inoculated seedlings, respectively (see Table S1 available as Supplementary data at *Tree Physiology* Online). When analyzed by RT-qPCR, *GLP1-7* had 1.96 and 1.54 times higher expression in the inoculated seedlings 7 and 90 DPI, respectively (Figure 5). By in situ hybridization, a remarkably high expression of *GLP1-7* was observed in the apical meristems and needles of inoculated seedlings (Figure 8A and B), compared with a strong expression in control seedling meristems (Figure 8C) and low or undetected expression in the control needles (Figure 8D). There was no hybridization of the sense probe observed in the inoculated seedling needle tissue (Figure 8E). The gene *BX253726* similar with ALE2 kinase (*AT2G20300*) had increased expression by 1.78 logFC (*P* < 0.01 and adj. *P* < 0.05), and the gene *BX682957*, which is similar with Embryo Defective 1270 (EMB1270) (*AT3G18110*), had increased expression by 0.94 logFC (*P* < 0.05) in the inoculated seedlings when compared with controls (see Table S1 available as Supplementary data at *Tree Physiology* Online). The gene *CR392068,* similar with Altered meristem program 1 protein (AMP1) (*AT3G54720*), was downregulated in the *M. extorquens*-inoculated seedlings by −0.98 logFC (*P* < 0.01) (see Table S1 available as Supplementary data at *Tree Physiology* Online).

### Flower development

The gene *BX681904*, similar with Terminal Flower 1 (TFL1) (*AT5G03840*), had 0.92 logFC (*P* < 0.05) higher expression in the *M. extorquens*-inoculated seedlings than in controls (see Table S1 available as Supplementary data at *Tree Physiology* Online). In contrast, the gene *AL750172* that is similar with agamous-like 8 (AGL8) transcription factor (*AT5G60910*) had decreased expression by −0.69 logFC (*P* < 0.05) in the inoculated seedlings (see Table S1 available as Supplementary data at *Tree Physiology* Online). Genes *CR392399* and *BX681731* were similar with Pollen Ole e1 allergen protein (*AT5G15780*), and both had reduced expression by −0.65 logFC (*P* < 0.05, *P* < 0.01, respectively) in the inoculated seedlings (see Table S1 available as Supplementary data at *Tree Physiology* Online). There were also two genes (*BX678708* and *BX254339*) similar with plantacyanins (*AT2G02850*, *AT3G26618*), which had reduced expression by −0.75 (*P* < 0.01 and adj. *P* < 0.01) and −0.49 logFC (*P* < 0.01), respectively, in the inoculated pine seedlings (see Table S1 available as Supplementary data at *Tree Physiology* Online). Other interesting downregulated genes in the inoculated seedlings were *BX677163* similar with nucleoredoxin (NRX1) (*AT1G60420*) by −0.57 logFC (*P* < 0.01), and *CR394036* similar with Jacalin-related lectin 32 (*AT3G16440*), by −0.81 logFC (*P* < 0.05; see Table S1 available as Supplementary data at *Tree Physiology* Online).

### Leaf and root development

The gene *BX680644*, similar with a CO_2_ response secreted protease (CRSP) (*AT1G20160*), required for stomata development, had increased expression by 0.97 logFC (*P* < 0.05), and the gene *BX680490* similar with LRR-type receptor protein kinase (RFGR1) (*AT3G24240*), which is required for proper root growth and development, had 0.88 logFC expression (*P* < 0.01) in the *M. extorquens*-inoculated seedlings compared with the controls (see Table S1 available as Supplementary data at *Tree Physiology* Online). In contrast, *BX250614*, resembling EXORDIUM like 3 (EXL3) (*AT5G51550*) had −0.65 logFC expression (*P* < 0.01 and adj. *P* < 0.01), and *BX784084* similar with the phosphatidylinositol transporter COW1 (*AT4G34580*) had −0.77 logFC expression (*P* < 0.05) in the inoculated seedlings compared with controls (see Table S1 available as Supplementary data at *Tree Physiology* Online). The gene *BX253632* similar with Gibberellin-regulated family protein GASA7 (*AT2G14900*), also attributable to gibberellin signaling, had −0.64 logFC expression (*P* < 0.01), and *BX679532*, a rapid alkalinization factor-like protein 34 (RALFL34) (*AT5G67070*), had −0.71 logFC expression (*P* < 0.05) in the inoculated seedlings when compared with the controls (see Table S1 available as Supplementary data at *Tree Physiology* Online).

### Defense

There were several pathogenesis-responsive (PR) genes activated due to *M. extorquens* DSM13060 inoculation. The gene *CR394078*, similar with Beta-1,3-glucanase (PR-2) (*AT4G16260*), was induced by 1.05 logFC (*P* < 0.01 and adj. *P* < 0.01). *AL750218* similar with Chitinase IV (PR-4) (*AT3G54420*) had increased expression by 1.61 logFC (*P* < 0.01), and *BX680492* similar with Thaumatin-like protein (PR-5) (*AT4G11650*), had 1.99 logFC expression (*P* < 0.01 and adj. *P* < 0.01) in the inoculated seedlings (see Table S1 available as Supplementary data at *Tree Physiology* Online). When studied by RT-qPCR, *PR-5* had slightly elevated expression, not statistically significant, in the inoculated seedlings 7 DPI, but a highly induced expression of 7.33-fold at 90 DPI (Figure 5). Other interesting upregulated genes associated with defense include *BX253434* similar to Powdery Mildew Resistant 5 (PMR5) protein (*AT5G58600*) (2.29 logFC, *P* < 0.05), *BX249433* similar with EF-Tu receptor (*AT5G20480*) (1.19 logFC, *P* < 0.01), *BX681871* similar with Annexin 1 (ANN1) (*AT1G35720*) (1.12 logFC), and *BX252125* similar with Activated Disease Resistance-Like 1 (ADR1-L1) (*AT4G33300*) (0.68 logFC, *P* < 0.05) (see Table S1 available as Supplementary data at *Tree Physiology* Online). However, there were also downregulated genes associated with defense in the inoculated seedlings, such as the gene *AL749574* similar with the disease resistance protein NDR1/HIN1-LIKE 3 (NHL3) (*AT5G06320*) (−1.01 logFC, *P* < 0.01), and *BX252568* similar with another disease resistance protein (*AT1G59620*) (−0.56 log FC, *P* < 0.01; see Table S1 available as Supplementary data at *Tree Physiology* Online).

### Stress responses

The gene *BX249322* similar with Tubulin 8 (*AT5G23860*) had increased expression in the *M. extorquens*-inoculated seedlings by 1.00 logFC (*P* < 0.01; see Table S1 available as Supplementary data at *Tree Physiology* Online). In addition, *BX678105* similar with Myo-Inositol Oxygenase 1 (MIOX1) (*AT1G14520*), also attributable to carbohydrate metabolism, had 0.81 logFC expression (*P* < 0.01), and *AL749806* similar with heat shock protein 81.4 (*AT5G56000*) associated with protein folding, had 0.48 logFC expression (*P* < 0.01; see Table S1 available as Supplementary data at *Tree Physiology* Online). There were also two genes, *BX678097* and *BX254612*, similar with chaperones DNA J protein C23 (DJC23) (*AT4G36040*) and HSP20-like superfamily protein (*AT1G07400*), which were upregulated by 0.69 and 0.50 logFC (*P* < 0.05), respectively, in the inoculated seedlings (see Table S1 available as Supplementary data at *Tree Physiology* Online). The infection by *M. extorquens* DSM13060 suppressed several stress response genes, such as *AL750772* similar with yeast autophagy 18 D-like protein (*AT3G56440*), by −0.99 logFC (*P* < 0.05), and *BX253464,* similar with CAD1 MAC/Perforin domain-containing protein (*AT1G29690*), by −0.59 logFC (*P* < 0.05; see Table S1 available as Supplementary data at *Tree Physiology* Online). There were also two genes, *AL749722* and *BX252163*, similar with a protein of hydroxyproline-rich glycoprotein family (EULS3) (*AT2G39050*), which had −0.65 (*P* < 0.01) and −0.49 logFC (*P* < 0.05) expression, respectively (see Table S1 available as Supplementary data at *Tree Physiology* Online). Other interesting downregulated genes associated with stress responses were *BX666019* similar with a heat-shock protein 21 (HSP21) (*AT4G27670*), by −0.65 logFC (*P* < 0.05), *BX680154* similar with Calmodulin-binding protein 25 (CAMBP25) (*AT2G41010*), by −0.51 logFC (*P* < 0.05) and *BX680071* similar with MYB domain protein R1 (MYBR1) (*AT5G67300*), by −0.60 logFC (*P* < 0.01; see Table S1 available as Supplementary data at *Tree Physiology* Online).

### Senescence and cell death

Several genes associated with senescence and cell death were downregulated in the *M. extorquens*-inoculated seedlings compared with controls. These were *AL750591*, similar to xyloclucan endotransglucosylase/hydrolase 24 (MERI-5) (*AT4G30270*) by −0.63 logFC (*P* < 0.05), *BX677134* similar with neurogenic locus notch-like protein (*AT4G14746*) by −0.91 logFC (*P* < 0.01 and adj. *P* < 0.01), *BX68049* similar with telomere repeat-binding factor (TRF5) (*AT1G72740*), by −0.52 logFC (*P* < 0.01 and adj. *P* < 0.01) and *CR392761*, similar with Chlorophyllase (CLH2) (*AT5G43860*), by −0.76 logFC (*P* < 0.01 and adj. *P* < 0.01; see Table S1 available as Supplementary data at *Tree Physiology* Online). When expression of *CLH2* was analyzed by RT-qPCR, the expression was −1.25 times lower in the inoculated seedlings than in controls at 90 DPI (Figure 5).

### Primary and secondary metabolism

There was one gene associated with Calvin cycle or glycolysis, *BX680264* similar with aldolase superfamily protein (FBA4) (*AT5G03690*), which had increased expression by 0.59 logFC (*P* < 0.01) in the *M. extorquens* inoculated seedlings (see Table S1 available as Supplementary data at *Tree Physiology* Online). Several genes on the chorismic acid pathway were differentially expressed. *BX677095* similar with 3-deoxy-d-arabino-heptulosonate-7-phosphate 2 (DAHP2) (*AT4G33510*) had increased expression by 0.56 logFC (*P* < 0.01) (see Table S1 available as Supplementary data at *Tree Physiology* Online). *BX681579* similar with 3-deoxy-d-arabino-heptulosonate 7-phosphate synthase (DHS2) (*AT4G33510*) and *BX253603* similar with chorismate mutase 3 (CM3) (*AT3G29200*) both had reduced expression by −0.49 (*P* < 0.01) and − 0.54 logFC (*P* < 0.05), respectively, in the inoculated seedlings compared with controls (see Table S1 available as Supplementary data at *Tree Physiology* Online).

The infection by *M. extorquens* DSM13060 induced expression of secondary metabolism genes. There was one cytochrome P450-like gene, *BX682159*, similar with CYP71 (*AT5G07990*), which had increased expression by 0.61 logFC (*P* < 0.01) in the *M. extorquens*-inoculated seedlings when compared with controls (see Table S1 available as Supplementary data at *Tree Physiology* Online). The RT-qPCR analysis confirmed a 1.81-fold increased expression for *CYP71* 90 DPI in the inoculated seedlings (Figure 5), and the gene expression was localized by in situ hybridization in the tissues of apical meristem of inoculated seedlings (Figure 8F), whereas there was no expression in the needles (Figure 8G) or in control meristems (Figure 8H). *CR393796* similar with AT2G24210 responsible for monoterpene synthesis was induced by 1.05 logFC (*P* < 0.05), *CR394028* similar with phenylalanine ammonia-lyase 2 (PAL2) (*AT3G53260*) by 0.49 logFC (*P* < 0.05) and *AL749926* similar with dihydroflavonol 4-reductase (DFR) (*AT5G42800*) by 1.23 logFC (*P* < 0.05) (see Table S1 available as Supplementary data at *Tree Physiology* Online). Furthermore, *BX249246,* similar with Laccase (*AT5G05390*), had a 0.99 logFC expression (*P* < 0.05) in the inoculated seedlings compared with controls (see Table S1 available as Supplementary data at *Tree Physiology* Online). The secondary metabolism was suppressed by downregulation of the gene *BX679131*, similar with leucoanthocyanidin dioxygenase (LDOX) (*AT4G22880*), by −0.55 logFC (*P* < 0.05; see Table S1 available as Supplementary data at *Tree Physiology* Online).

Metabolite level analysis of pine seedlings showed that there were 12 significant differences in the case of needles, 11 significant differences in the case of roots, and 16 significant differences in the case of stems (Table 2). A considerable number of metabolites also showed ‘high variable importance in projection’ according to the PLS-DA (Table 2). Specific metabolites with significant differences could be identified in the needles. Quantities of galactose methoxyate (*P* < 0.05) and methyl inositol (*P* < 0.01) were significantly lower, whereas those of sucrose (*P* = 0.062), ononitol (*P* < 0.05) and malic acid (*P* = 0.06) were higher in the infected seedlings than in controls (Table 2).

**Table 2:** Metabolites with significant (*P* < 0.1, fold change >2 or <0.5) differences when comparing treated (inoculated with *Methylorubrum extorquens* DSM13060) and untreated plants

Retention time index	Fragment used for quantification	Fold change (log2)	*P*-value	High variable importance in PLS-DA projection	Metabolite identity
**Needles**					
800	59	−1.09	0.099		Known unknown
1319	176	1.59	0.060		Malic acid
1690	450	−1.73	0.028		
1698	348	1.10	0.087		
1709	206	−3.12	1.97e−06	1	Methyl inositol
1738	317	−1.24	0.0065		
1777	376	−2.29	0.023		
1796	319	−1919	0.027	1	Galactose methoxyate
1816	391	5.06	1.03e−10	1	
1840	147	1.60	0.030	1	Ononitol
2070	100	1.16	0.027		
2415	107	1.01	0.062		Sucrose
**Roots**					
507	156	−1555	0.0076	1	
800	130	3.48	0.033	1	
914	61	−1.68	0.087		
1016	212	1.06	0.070		
1075	190	−1.43	0.099		
1319	335	1.42	0.013	1	
1535	231	1.27	0.098		
1597	175	−1.53	0.087		
1689	258	1.72	0.023		
2070	142	−1.39	0.026		
2415	263	1.79	0.0060	1	
**Stem**					
442	141	−1.32	0.0046	1	
464	100	−1.24	0.028	1	
489	146	−3.58	0.021		
490	45	−5.05	0.019	1	
493	156	−2.86	0.018		
530	40	−4.16	0.017	1	
800	105	−1.10	0.085		
1340	155	−2.51	0.036	1	
1535	141	4.96	0.0050	1	
1597	74	−1.08	0.017	1	
1698	301	2.15	0.034		
1709	206	−1.12	0.0066	1	
1816	204	7.21	1.24e−05	1	
1840	308	2.15	0.034		
1841	150	1.29	0.042		
2415	378	1.23	0.00089		

## Discussion

The stringent treatments prior to inoculation, heat treatment combined with efficient surface sterilization, ensured that the pine seeds were cured of the majority of innate microbiota. The heat treatment is reported to remove 70–90% of microbes (Holland and Polacco 1992), and according to our earlier tests, heat-treated, surface-sterilized pine seedlings lack *M. extorquens* DSM13060 (data not shown). The inoculated pine seedlings were harvested at 90 DPI, as the endophytic interaction with pine host is well established at this time point and *M. extorquens* DSM13060 colonizes the pine seedlings systemically, being found in all plant tissues from roots to the apical shoot tips (Figure 1, Koskimäki et al. 2021). The colonized seedlings have higher biomass, i.e., induced growth of both shoots and roots, than the heat-treated controls (Pohjanen et al. 2014). *M. extorquens* DSM13060 is an intracellular colonizer, often aggregating around host nucleus (Koskimäki et al. 2015). In the current study, the infection by *M. extorquens* DSM13060 affected cell wall biosynthesis genes of the host, which most likely reflects colonization of cell interiors by this bacterium. The genes *CGR2*, *XTH9* and *HXXXD*-type acyl-transferase had lower expression in the inoculated seedlings. CGR2 is involved in homogalacturonan methylesterification in the Golgi apparatus (Held et al. 2011, Kim et al. 2015), XTH9 cleaves and rebuilds xyloglucan polymers, affecting both primary and secondary cell wall structure (Kushwah et al. 2020) and HXXXD-type acyl- transferase is involved in suberin biosynthesis of the cell wall (Gou et al. 2009).

Because we had isolated RNA from whole seedlings for transcriptional profiling, we performed in situ hybridization to localize expression of selected genes within pine tissues. The genes upregulated in the inoculated seedlings were highly expressed specifically in the shoot apical meristem, more rarely in the needles, and not in stem or root tissues. The gene expression analysis suggested that *M. extorquens* DSM13060 activates auxin- and ET-associated hormonal pathways, and possibly suppresses salicylic (SA) and abscisic acid (ABA) signaling while infecting the host *P. sylvestris*. Ethylene is recognized in plants by a family of membrane-bound receptors, such as *ETR2* that was upregulated, which then control the expression of *ERF* genes (Chen et al. 2005). *ERF3*, which is an ERF-B6 type transcription factor, and *ERF4*, which is an ERF-B1 type transcription factor (Zeng et al. 2016), both had increased expression, whereas the transcriptional inhibitor DREB-A5 type ERF, *ERF010/DEAR2* (Zeng et al. 2016), had reduced expression in *M. extorquens*-inoculated seedlings. ERF3 modifies expression of *RABH1e* (Zeng et al. 2016), which was also upregulated in inoculated pine seedlings, and is involved in protein trafficking through the Golgi apparatus (Rehman and Di Sansebastiano 2014).

In general, the ET signaling pathway is activated as a response to several plant-associated microbes, both rhizospheric and endophytic ones (Ahn et al. 2007, Ardanov et al. 2011, Hardoim et al. 2015), to induce systemic resistance (ISR). Supporting this, a set of pathogenesis-responsive (PR) genes, *PR-2*, *PR-4* and *PR-5*, was upregulated in response to the *M. extorquens* infection in pine seedlings. However, the RT-qPCR study showed that *PR-5* was not induced at the beginning of infection, but only at the later stage of 90 DPI. Expression of several *Plasmodesmata-located proteins* (*PDLP8*) and simultaneous downregulation of several *Lipid Transfer Proteins* (*LTP*), which occurred in the inoculated seedlings, is indicative of repression of the SA-mediated systemic acquired response (SAR) (Carella et al. 2015). Furthermore, *ATGRP23* and *AD1 MAC/Perforin domain-containing protein*, which are induced by abscisic and salicylic acid stimuli (Morita-Yamamuro et al. 2005, Park et al. 2008), were downregulated, providing further evidence of SAR repression in the inoculated pine seedlings.

Similar to ET, there were several genes on the auxin signaling pathway differentially expressed in the *M. extorquens*-inoculated pine seedlings. For example, the auxin-response factor *ARF2* was upregulated in the inoculated seedlings. ARFs are transcriptional factors that bind to the auxin-responsive promoter elements of downstream transcription factors (Lim et al. 2010), such as *MYB44*, which was downregulated in the inoculated pine seedlings. In Arabidopsis, ARF2 is responsible for leaf longevity and suppressing senescence-associated gene expression (Lim et al. 2010). *CUL1*, which had higher expression in the inoculated seedlings, is also associated with the auxin signaling pathway as a component of the E3 ubiquitin ligase SCF^TIR1^ complex. Higher expression of *CUL1* renders more AUX/IAA repressors to the ubiquitin degradation pathway and induces expression of auxin-responsive genes (Gray et al. 1999, Ren et al. 2005).

Auxin biosynthesis genes were not upregulated in pine seedlings inoculated with *M. extorquens* DSM13060, and the bacterium does not produce auxins (Pirttilä et al. 2004, Koskimäki et al. 2015). However, the CUL1 protein is modified by beneficial bacteria in human gut epithelial cells (Collier-Hyams et al. 2005) through butyrate-induced neddylation (Kumar et al. 2007), which could be employed as a mechanism of manipulating the pine host by *M. extorquens* DSM13060. On the other hand, *M. extorquens* DSM13060 genome possesses several copies of the *phospholipase (PLA2)* gene (Koskimäki et al. 2015). In general, PLA_2_ enzymes catalyze the hydrolysis of membrane phospholipids, producing free fatty acids and lysophospholipids (Chen et al. 2011). PLA_2_ enzymes and their enzymatic products are implicated in a range of cellular processes in plants, such as plant growth, development, stress responses, defense signaling (Chapman 1998, Chen et al. 2011, Polkowska-Kowalczyk et al. 2011) and, specifically, auxin signal transduction (Scherer et al. 2007). In Arabidopsis, PLA_2_ contributes to cell elongation and shoot gravitropism via auxin signaling, including the ubiquitin proteolysis (E3 ubiquitin ligase SCF^TIR1^ complex) pathway (Lee et al. 2003, Scherer et al. 2007).

A third potential mechanism of affecting the auxin signaling pathway of pine by *M. extorquens* DSM13060 is through induction of *HYL1*. The *HYL1 gene* encodes a double-stranded RNA binding protein, which in general has an important role in miRNA and siRNA processing. This protein affects plant development through auxin, cytokinin and abscisic acid signaling (Lu and Fedoroff 2000, Vazquez et al. 2004). Although miRNA and siRNA are indicated to play a role in plant–microbe interactions (Katiyar-Agarwal et al. 2007, Thiebaut et al. 2014), *M. extorquens* DSM13060 possesses at least two successive genes annotated as microRNA 790 (mir-790, JGI IDs *2507324312, 2507324313*). The first one, *2507324312* has similarity with *miR5554a* of alfalfa (*Medicago truncatula*), associated with drought stress (Wang et al. 2011). Furthermore, it shares similarity with *WD-repeat protein-like* of *P. pinaster* (DR092239) that corresponds to Arabidopsis ATWDR5a (AT3G49660), which is associated with auxin signaling and root growth (Liu et al. 2018). The second one, *2507324313*, has similarity with *Caenorhabditis elegans* miR-790 stem-loop and mature miRNA structures, which have a potential target site in 60S ribosomal protein L10 of *P. pinaster* (TC166703). To enable miRNA delivery to the host cell, *M. extorquens* DSM13060 carries genes for type-I, type-II and type-IV (T4SS) secretion systems (Koskimäki et al. 2015). The *T4SS* has sequence similarity with *dot/icm system* of the intracellular human pathogen *Legionella*, which enables bacterial delivery of effector molecules, genetic exchange and intracellular replication during invasion of eukaryotic cells (Brüggemann et al. 2006).

**Figure 9.**
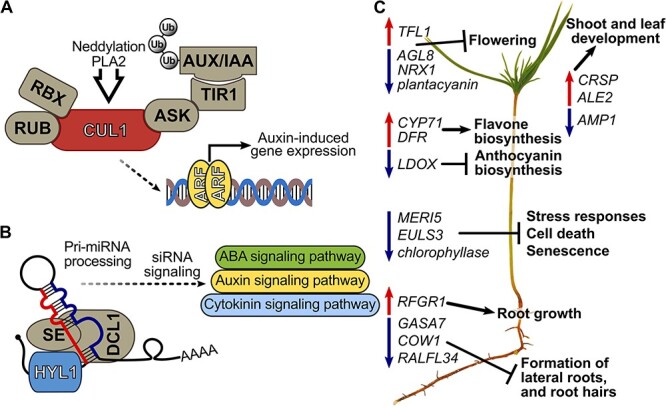
Proposed key mechanisms of *Methylorubrum extorquens* DSM13060 in affecting hormonal signaling of Scots pine and subsequent gene networks. (A) the bacterium induces host *CUL1*, potentially through phospholipase A2 enzymes or neddylation, which activates auxin signaling by submitting the AUX/IAA repressor to the ubiquitin degradation pathway. (B) Bacterial induction of *HYL1* can affect auxin, abscisic acid (ABA) and cytokinin signaling via miRNA and siRNA processing. (C) Developmental genes behind flowering, root hair and lateral root formation are repressed, whereas genes for shoot and root growth are induced in pine seedlings. Biosynthesis genes of flavones are induced, and anthocyanin synthesis genes are repressed. Decreased expression of genes involved in senescence and cell death occurs in colonized seedlings potentially due to production of methyl-esterified 3-hydroxybutyrate oligomers (Koskimäki et al. 2016) and ononitol. ALE2 = abnormal leaf shape 2, ARF = auxin-responsive factor, ASK = apoptosis signal-regulating Kinase-1, AUX/IAA = auxin/indole acetic acid, COW1 = phosphatidylinositol transporter, CRSP = CO_2_ response secreted protease, CUL1 = Cullin-1, DCL = dicer-like 1, Pri-miRNA = primary transcript micro-RNA, RBX = RING H2-type domain, RUB = related to ubiquitin, SE = serrate protein, Ub = ubiquitin.

Potentially through the changes in hormonal signaling, expression of a number of developmental genes was altered in *M. extorquens*-inoculated pine seedlings. The germin-like protein, GLP1-7, which had high expression in inoculated seedlings, is regulated by auxin in *P. salicina* (El-Sharkawy et al. 2010). Similarly, *ALE2*, upregulated in the inoculated seedlings, is controlled by auxin and required for differentiation and morphogenesis of epidermal cells in the protoderm of a forming embryo in Arabidopsis (Tanaka et al. 2007). Development of reproductive organs was mainly repressed in the inoculated seedlings, indicated by upregulation of *TFL1*, the repressor of flower identity (Baumann et al. 2015) and simultaneous downregulation of flower developmental genes *AGL8* (Pnueli et al. 1994), *O-glucosyl hydrolases family protein* (involved in anther development [Balasubramanian et al. 2012]), *plantacyanin*, *Pollen Ole e1 allergen* and *NRX1* (all involved in pollen tube growth and pollen function [Alché et al. 2004, Dong et al. 2005, Qin et al. 2009]) and *Jacalin-related lectin 32* (needed for female gametophyte development [Pagnussat et al. 2005]). Indicative of suppression of root hair and lateral root formation, *GASA7*, *COW1* and *RALFL34* (Pitts et al. 1998, Zhang and Wang 2008, Murphy et al. 2016) had low expression in the *M. extorquens*-inoculated seedlings. Concurrently, *RFGR1*, the candidate receptor for root growth factor inducing root elongation (Shinohara et al. 2016) was upregulated in the inoculated seedlings, which do exhibit significantly higher root biomass than controls already 60 DPI in vitro (Pohjanen et al. 2014).

The changes in hormonal signaling pathways of the seedlings inoculated with *M. extorquens* DSM13060 potentially also altered the secondary metabolism of pine. The upregulated biosynthesis pathways of monoterpenes (Nissen et al. 2010), and laccase, involved in lignin formation (Gavnholt and Larsen 2002), can be linked with resistance induction. An interesting feature was the induction of flavonol and flavone biosynthesis through upregulation of *CYP71* and *DFR,* and downregulation of anthocyanin biosynthesis (*LDOX*) by the bacterium. These changes may be the result of hormonal signaling induced by the infection, as anthocyanins accumulate in many plant species in response to ABA, but their biosynthesis is repressed by ET (Das et al. 2012), which together with auxin induces flavonol accumulation (Lewis et al. 2011).

The infection by *M. extorquens* DSM13060 reduced activity of genes associated with senescence and cell death, *MERI5* (Shi et al. 2015)*, CLH2* (Berardini et al. 2015), as well as *yeast autophagy 18 D-like protein* (Xiong et al. 2005). An interesting gene associated with cell longevity is the telomere repeat binding factor (*TRF5*), which had reduced expression in the inoculated seedlings. Although TRFs are generally needed for maintenance of telomeres (Procházková Schrumpfová et al. 2014), *TRF5* of Arabidopsis controls growth-related processes through organization of membrane components and RNA processing in mitochondria (Meng et al. 2019). The positive effects on senescence and cell death by *M. extorquens* DSM13060 could be due to polyhydroxybutyrate-producing capacity of this bacterium. We have earlier discovered that *M. extorquens* DSM13060 produces high quantities of methyl-esterified 3-hydroxybutyrate (ME-3HB) oligomers, which possess strong antioxidant activity towards hydroxyl radical (Koskimäki et al. 2016). The monomer, 3-hydroxybutyric acid (HBA), inhibits apoptosis under glucose deprivation, balances mitochondrial homeostasis and rescues activities of mitochondrial respiratory chain complexes in animal cells (Tieu et al. 2003, Maalouf et al. 2007, Zhang et al. 2013), and could play similar roles in the plant cell as well.

The ME-3HB oligomers could also be responsible for suppressing stress responses in the inoculated pine seedlings. For example, genes for glyoxal oxidase-related protein (Daou and Faulds 2017), hydroxyproline-rich protein (EULS3) (Van Hove et al. 2014) and heat-shock protein 21 (Zhong et al. 2013) were downregulated in inoculated seedlings. The ME-3HB oligomers can effectively compensate for loss of glutathione *S*-transferase enzymes in yeast and protect pine cells against oxidative stress in vitro (Pirttilä et al. 2004, Koskimäki et al. 2016). Downregulation of genes for hydroxyproline-rich proteins suggests that the inoculated seedlings were not suffering from osmotic stress. *Myo-Inositol oxygenase 1*, responsible of myo-inositol degradation, was upregulated, and the metabolic profile revealed that the seedlings had reduced quantities of galactosyl and methyl inositol (pinitol), and increased quantities of ononitol. Pinitol is associated predominantly with overcoming abiotic stress (Chiera et al. 2006, Sengupta et al. 2015), ononitol being the intermediate of pinitol from myo-inositol (Chiera et al. 2006) and the starting compound for biosynthesis of galatosyl ononitol (Peterbauer and Richter 1998). Methylated inositols are abundant in legumes (Phillips and Smith 1974), and production of ononitol and *O*-methyl-scyllo-inositol has been reported in *Rhizobium leguminosarum* (Skøt and Egsgaard 1984), indicating that ononitol could be a bacterial product. The inositol methyltransferase (IMT, EC 2.1.1.129), responsible for ononitol synthesis, is characterized so far only in a couple of plant species (Chiera et al. 2006, Sengupta et al. 2008) and not in bacteria, but we found several candidate genes for IMT in the *M. extorquens* DSM13060 genome (data not shown).

There were also elevated levels of sucrose and malic acid in the needles of inoculated seedlings. Enhanced photosynthesis was not observed by gene expression analysis, which suggests that increased sucrose levels resulted from starch degradation. *M. extorquens* DSM13060 is not capable of utilizing sucrose as a carbon source (Pirttilä et al. 2000), therefore the elevated sucrose levels were unlikely to benefit the bacterium. In contrast, malic acid is a compound luring the beneficial soil bacteria into the plant root system (Rudrappa et al. 2008, Yuan et al. 2015), the most important tricarboxylic acid in the rhizobial symbiosis (Mitsch et al. 2018), and a preferred substrate for *M. extorquens* DSM13060 (Pirttilä et al. 2000). Therefore, we suggest that malic acid could play an important role in pine—*M. extorquens* interaction. To elucidate the interaction further, deletion mutants of both counterparts and fluorescent tags controlled by specific gene promoters could reveal interesting insights into the endosymbiosis of Scots pine trees.

### Concluding remarks

Our study reveals various pathways employed by the endosymbiont *M. extorquens* DSM13060 to affect development and stress tolerance of *P. sylvestris* (Figure 9). The key mechanisms affecting hormonal signaling of the host can take place through bacterial phospholipase A2 enzymes, neddylation of CUL1 and miRNA processing through *HYL1*. As a result, developmental genes behind flowering and formation of root hairs and lateral roots become repressed, and genes responsible for shoot and root growth are induced. The secondary metabolism genes of flavone and flavonol biosynthesis are activated, whereas those on the anthocyanin pathway are repressed. Colonization by the endosymbiont results in decreased expression of genes involved in senescence and cell death. Production of ME-3HB oligomers by *M. extorquens* and biosynthesis of cyclitols could aid the host in tolerance of biotic and abiotic stress.

## Data and materials availability

The gene expression data has been submitted to Genbank’s GEO (https://www.ncbi.nlm.nih.gov/geo/info/spreadsheet.html) under the accession no. GSE170995.

## Supplementary Material

TableS1_Koskimakietal_tpab102
