# Supplementary material for: The meristem-associated endosymbiont Methylorubrum extorquens DSM13060 reprograms development and stress responses of pine seedlings
Source: Tree Physiol. 2021 Jul 29;42(2):391–410. doi: 10.1093/treephys/tpab102 (PMC8842435; doi:10.1093/treephys/tpab102)
Supplement: TableS1_Koskimakietal_tpab102 [file tables1_koskimakietal_tpab102.pdf]

Gene ID	*Pinus taeda* MAKER gene model	Arabidopsis locus	Score	E value	Putative function/name	GO term	LOG Fold change	p value	Adj. p value
**Up-Regulated Genes**									
CR394337	PITA_000069518-RA	AT2G36970	263	6e-82	UDP-Glycosyltransferase superfamily protein	transferase activity, transferring hexosyl groups (GO:0016758); metabolic process (GO:0008152)	3.18	0.008305	0.295548
BX678370	PITA_000032855-RA	AT5G54580	123	8e-37	ORRM2, RNA-binding (RRM/RBD/RNP motifs) family protein	nucleotide binding (GO:0000166)	2.77	0.011748	0.326025
BX253434	PITA_000043948-RA	AT5G58600	364	1e-100	POWDERY MILDEW RESISTANT 5 (PMR5)	multi-organism process (GO:0051704); response to stimulus (GO:0050896); response to external biotic stimulus (GO:0043207); response to other organism (GO:0051707); response to stress (GO:0006950); response to biotic stimulus (GO:0009607); catalytic activity (GO:0003824)	2.29	0.042936	0.549758
AL749800	PITA_000018537-RA	AT1G09700	79	5e-15	HYPONASTIC LEAVES 1 (HYL1), nuclear dsRNA binding protein	response to stimulus (GO:0050896); organic substance metabolic process (GO:0071704); single-organism cellular process (GO:0044763); response to hormone (GO:0009725); negative regulation of biological process (GO:0048519); metabolic process (GO:0008152); single-organism process (GO:0044699); response to oxygen-containing compound (GO:1901700); response to endogenous stimulus (GO:0009719); single-organism developmental process (GO:0044767); multicellular organismal development (GO:0007275); developmental process (GO:0032502); response to chemical (GO:0042221); single-multicellular organism process (GO:0044707); primary metabolic process (GO:0044238); cellular response to	2.24	0.002078	0.14972

						stimulus (GO:0051716); multicellular organismal process (GO:0032501); response to acid chemical (GO:0001101); single-organism metabolic process (GO:0044710); catalytic activity (GO:0003824)			
CR393282	PITA_000010539-RA	AT3G05950	105	8e-23	Germin-like protein (GLP1-7)	apoplast (GO:0048046)	2.16	4.93E-05	0.015558
BX680492	PITA_000033081-RA	AT4G11650	177	8e-45	Osmotin-like protein (OSM34), putative pathogenesis-related protein (PR-5)	response to organic substance (GO:0010033); defense response to fungus, incompatible interaction (GO:0009817); multi-organism process (GO:0051704); innate immune response (GO:0045087); response to stimulus (GO:0050896); immune response (GO:0006955); immune system process (GO:0002376); single-organism cellular process (GO:0044763); response to hormone (GO:0009725); negative regulation of biological process (GO:0048519); regulation of response to stress (GO:0080134); single-organism process (GO:0044699); response to bacterium (GO:0009617); response to oxygen-containing compound (GO:1901700); multi-organism cellular process (GO:0044764); response to endogenous stimulus (GO:0009719); response to ethylene (GO:0009723); symbiosis, encompassing mutualism through parasitism (GO:0044403); interspecies interaction between organisms (GO:0044419); response to external biotic stimulus (GO:0043207); response to other organism (GO:0051707); regulation of defense response (GO:0031347); response to stress (GO:0006950); response to chemical (GO:0042221); response to	1.99	2.67E-10	1.9E-06

						biotic stimulus (GO:0009607); response to salt stress (GO:0009651); regulation of response to stimulus (GO:0048583)			
BX253726	PITA_000025472-RA	AT2G20300	667	0.0	ABNORMAL LEAF SHAPE 2 (ALE2), receptor-like protein kinase (RLK)	seed development (GO:0048316); metabolic process (GO:0008152); single-organism process (GO:0044699); reproductive structure development (GO:0048608); reproductive system development (GO:0061458); fruit development (GO:0010154): anatomical structure development (GO:0048856); single-organism developmental process (GO:0044767); post-embryonic development (GO:0009791); multicellular organismal development (GO:0007275); developmental process (GO:0032502); single-multicellular organism process (GO:0044707); embryo development ending in seed dormancy (GO:0009793); primary metabolic process (GO:0044238); embryo development (GO:0009790); multicellular organismal process (GO:0032501); catalytic activity (GO:0003824); carbohydrate derivative binding (GO:0097367); purine ribonucleoside triphosphate binding (GO:0035639); purine nucleoside binding (GO:0001883); purine ribonucleoside binding (GO:0032550); ribonucleoside binding (GO:0032549); nucleoside binding (GO:0001882); purine ribonucleotide binding (GO:0032555); purine nucleotide binding (GO:0017076); nucleotide binding (GO:0000166); nucleoside phosphate binding (GO:1901265); ribonucleotide binding (GO:0032553); ATP binding (GO:0005524); anion	1.78	0.000153	0.034101

						binding (GO:0043168); small molecule binding (GO:0036094); adenyl ribonucleotide binding (GO:0032559); adenyl nucleotide binding (GO:0030554); protein serine/threonine kinase activity (GO:0004674)			
BX679686	PITA_000013968-RA	AT3G15210	57	2e-07	ETHYLENE RESPONSE FACTOR (ERF4)	response to organic substance (GO:0010033); multi-organism process (GO:0051704); innate immune response (GO:0045087); response to stimulus (GO:0050896); immune response (GO:0006955); organic substance metabolic process (GO:0071704); immune system process (GO:0002376); negative regulation of ethylene-activated signaling pathway (GO:0010105); negative regulation of phosphorelay signal transduction system (GO:0070298); single-organism cellular process (GO:0044763); response to hormone (GO:0009725); negative regulation of biological process (GO:0048519); regulation of response to stress (GO:0080134); metabolic process (GO:0008152); regulation of ethylene-activated signaling pathway (GO:0010104); regulation of phosphorelay signal transduction system (GO:0070297); negative regulation of intracellular signal transduction (GO:1902532); jasmonic acid mediated signaling pathway (GO:0009867); cellular response to jasmonic acid stimulus (GO:0071395); single-organism process (GO:0044699); response to bacterium (GO:0009617); response to oxygen-containing compound (GO:1901700);	1.69	0.031388	0.489843

						response to endogenous stimulus (GO:0009719); ethylene-activated signaling pathway (GO:0009873); response to ethylene (GO:0009723); cellular response to ethylene stimulus (GO:0071369); response to external biotic stimulus (GO:0043207); response to other organism (GO:0051707); regulation of defense response (GO:0031347); response to stress (GO:0006950); response to jasmonic acid (GO:0009753); response to chemical (GO:0042221); response to biotic stimulus (GO:0009607); primary metabolic process (GO:0044238); phosphorelay signal transduction system (GO:0000160); activation of innate immune response (GO:0002218); activation of immune response (GO:0002253); cellular response to stimulus (GO:0051716); regulation of response to stimulus (GO:0048583); response to acid chemical (GO:0001101)			
BX250299	PITA_000041605-RA	AT1G78830	49	1e-06	Curculin-like (mannose-binding) lectin family protein	response to organic substance (GO:0010033); response to stimulus (GO:0050896); response to hormone (GO:0009725); response to endogenous stimulus (GO:0009719); response to chemical (GO:0042221); apoplast (GO:0048046)	1.64	0.013173	0.349164
AL750218	PITA_000019663-RA	AT3G54420	244	4e-65	Chitinase family protein (EP3), putative pathogenesis-related protein 4 (PR-4)	single organism reproductive process (GO:0044702); multi-organism process (GO:0051704); innate immune response (GO:0045087); response to stimulus (GO:0050896); immune response (GO:0006955); developmental process involved in reproduction (GO:0003006); organic substance metabolic process (GO:0071704); immune system process	1.61	5.02E-05	0.015558

						(GO:0002376); single-organism cellular process (GO:0044763); reproductive process (GO:0022414); seed development (GO:0048316); metabolic process (GO:0008152); single-organism process (GO:0044699); reproductive structure development (GO:0048608); reproductive system development (GO:0061458); response to bacterium (GO:0009617); fruit development (GO:0010154); anatomical structure development (GO:0048856); single-organism developmental process (GO:0044767); post-embryonic development (GO:0009791); multicellular organismal development (GO:0007275); response to external biotic stimulus (GO:0043207); response to other organism (GO:0051707); response to stress (GO:0006950); developmental process (GO:0032502); single-multicellular organism process (GO:0044707); response to biotic stimulus (GO:0009607); embryo development ending in seed dormancy (GO:0009793); primary metabolic process (GO:0044238); embryo development (GO:0009790); organic substance catabolic process (GO:1901575); cellular response to stimulus (GO:0051716); multicellular organismal process (GO:0032501); catalytic activity (GO:0003824); carbohydrate derivative binding (GO:0097367)			
CR394230	PITA_000065855-RA	AT5G56950	278	3e-92	NFA3/NAP1.3 histone chaperone	-	1.55	0.033366	0.497643
BX682105	PITA_000056514-RA	AT3G05060	628	1e-180	Putative SAR DNA-binding protein	organic substance metabolic process	1.50	0.002204	0.152627

						(GO:0071704); metabolic process (GO:0008152); primary metabolic process (GO:0044238)			
BX251681	PITA_000006558-RA	AT5G39190	97	2e-20	Germin-like protein (GLP2a)	apoplast (GO:0048046)	1.48	1.55E-07	0.000552
BX254421	PITA_000092224-RA	AT3G23410	588	1e-168	FATTY ALCOHOL OXIDASE 3 (FAO3)	response to stimulus (GO:0050896); organic substance metabolic process (GO:0071704); metabolic process (GO:0008152); single-organism process (GO:0044699); response to stress (GO:0006950); single-organism metabolic process (GO:0044710); catalytic activity (GO:0003824); nucleotide binding (GO:0000166); nucleoside phosphate binding (GO:1901265); anion binding (GO:0043168); small molecule binding (GO:0036094)	1.47	0.005264	0.231825
CR354631	PITA_000042735-RA	AT2G02040	861	0	ATPTR2-B, NTR1, PTR2-B, PTR2, ATPTR2 peptide transporter 2	membrane (GO:0016020); transporter activity (GO:0005215); oligopeptide transport (GO:0006857)	1.34	0.041681	0.541468
AL749926	PITA_000093713-RA	AT5G42800	173	1e-43	Putative Dihydroflavonol 4-reductase (DFR)	organic substance metabolic process (GO:0071704); metabolic process GO:0008152); single-organism process (GO:0044699); single-organism metabolic process (GO:0044710); catalytic activity (GO:0003824)	1.23	0.011338	0.326025
BX249433	PITA_000010198-RA	AT5G20480	263	4e-70	Putative EF-Tu receptor (EFR), receptor for bacterial PAMP (pathogen associated molecular patterns) EF-Tu	multi-organism process (GO:0051704): Innate immune response (GO:0045087); response to stimulus (GO:0050896); immune response (GO:0006955); organic substance metabolic process (GO:0071704); immune system process (GO:0002376); single-organism cellular process (GO:0044763); regulation of response to stress (GO:0080134); metabolic process (GO:0008152); single-organism process (GO:0044699);	1.19	0.001388	0.117623

						response to bacterium (GO:0009617); response to external biotic stimulus (GO:0043207); response to other organism (GO:0051707); regulation of defense response (GO:0031347); response to stress (GO:0006950); response to biotic stimulus (GO:0009607); primary metabolic process (GO:0044238); activation of innate immune response (GO:0002218); activation of immune response (GO:0002253); cellular response to stimulus (GO:0051716); transmembrane receptor protein tyrosine kinase signaling pathway (GO:0007169); regulation of response to stimulus (GO:0048583); enzyme linked receptor protein signaling pathway (GO:0007167); catalytic activity (GO:0003824); carbohydrate derivative binding (GO:0097367); purine ribonucleoside triphosphate binding (GO:0035639); purine nucleoside binding (GO:0001883); purine ribonucleoside binding (GO:0032550); ribonucleoside binding (GO:0032549); nucleoside binding (GO:0001882); purine ribonucleotide binding (GO:0032555); purine nucleotide binding (GO:0017076); nucleotide binding (GO:0000166); nucleoside phosphate binding (GO:1901265); ribonucleotide binding (GO:0032553); ATP binding (GO:0005524); anion binding (GO:0043168); small molecule binding (GO:0036094); adenyl ribonucleotide binding (GO:0032559); adenyl nucleotide binding			

						(GO:0030554); protein serine/threonine kinase activity (GO:0004674)			
BX682647	PITA_000074847-RA	AT3G56080	29	0.63	S-adenosyl-L-methionine-dependent methyltransferases superfamily protein PMT22	metabolic process (GO:0008152); catalytic activity (GO:0003824)	1.13	0.013828	0.353479
BX681871	PITA_000053311-RA	AT1G35720	216	3e-56	Putative Annexin 1 (ANN1), ROS-regulated Ca2+ transport protein	response to organic substance (GO:0010033); single organism reproductive process (GO:0044702); tissue development (GO:0009888); response to stimulus (GO:0050896); developmental process involved in reproduction (GO:0003006); single-organism cellular process (GO:0044763); response to hormone (GO:0009725); reproductive process (GO:0022414); cellular process involved in reproduction in multicellular organism (GO:0022412); seed development (GO:0048316); single-organism process (GO:0044699); reproductive structure development (GO:0048608); reproductive system development (GO:0061458); response to oxygen-containing compound (GO:1901700); fruit development (GO:0010154); anatomical structure development (GO:0048856); response to endogenous stimulus (GO:0009719); multicellular organism reproduction (GO:0032504); seed trichome elongation (GO:0090378); single-organism developmental process (GO:0044767); post-embryonic development (GO:0009791); multicellular organismal development (GO:0007275); response to stress (GO:0006950); developmental process	1.12	0.048805	0.57153

						(GO:0032502); response to chemical (GO:0042221); single-multicellular organism process (GO:0044707); seed trichome differentiation (GO:0090376); response to salt stress (GO:0009651); multicellular organismal process (GO:0032501); response to acid chemical (GO:0001101); plant epidermis development (GO:0090558); apoplast (GO:0048046); catalytic activity (GO:0003824); carbohydrate derivative binding (GO:0097367); purine ribonucleoside triphosphate binding (GO:0035639); purine nucleoside binding (GO:0001883); purine ribonucleoside binding (GO:0032550); ribonucleoside binding (GO:0032549); nucleoside binding (GO:0001882); purine ribonucleotide binding (GO:0032555); purine nucleotide binding (GO:0017076); nucleotide binding (GO:0000166); nucleoside binding (GO:0000166); nucleoside phosphate binding (GO:1901265); ribonucleotide binding (GO:0032553); ATP binding (GO:0005524); anion binding (GO:0043168); small molecule binding (GO:0036094); adenyl ribonucleotide binding (GO:0032559); adenyl nucleotide binding (GO:0030554)			
BX680819	PITA_000082701-RA	AT1G11050	40	0.002	Protein kinase superfamily protein	organic substance metabolic process (GO:0071704); metabolic process (GO:0008152); primary metabolic process (GO:0044238); catalytic activity (GO:0003824); carbohydrate derivative binding (GO:0097367); purine ribonucleoside triphosphate binding (GO:0035639); purine nucleoside binding (GO:0001883); purine ribonucleoside binding (GO:0032550);	1.09	2.35E-05	0.009311

						ribonucleoside binding (GO:0032549); nucleoside binding (GO:0001882); purine ribonucleotide binding (GO:0032555); purine nucleotide binding (GO:0017076); nucleotide binding (GO:0000166); nucleoside phosphate binding (GO:1901265); ribonucleotide binding (GO:0032553); ATP binding (GO:0005524); anion binding (GO:0043168); small molecule binding (GO:0036094); adenyl ribonucleotide binding (GO:0032559); adenyl nucleotide binding (GO:0030554); protein serine/threonine kinase activity (GO:0004674)			
BX680159	PITA_000003774-RA	AT5G08610	621	1e-178	Putative Pigment Defective 340 (PDE340), DEAD-box RNA helicase	organic substance metabolic process (GO:0071704); metabolic process (GO:0008152); primary metabolic process (GO:0044238); catalytic activity (GO:0003824);); carbohydrate derivative binding (GO:0097367); purine ribonucleoside triphosphate binding (GO:0035639); purine nucleoside binding (GO:0001883); purine ribonucleoside binding (GO:0032550); ribonucleoside binding (GO:0032549); nucleoside binding (GO:0001882); purine ribonucleotide binding (GO:0032555); purine nucleotide binding (GO:0017076); nucleotide binding (GO:0000166); nucleoside phosphate binding (GO:1901265); ribonucleotide binding (GO:0032553); ATP binding (GO:0005524); anion binding (GO:0043168); small molecule binding (GO:0036094); adenyl ribonucleotide binding (GO:0032559); adenyl nucleotide binding (GO:0030554)	1.08	0.016793	0.392682

CR394078	PITA_000018769-RA	AT4G16260	297	1e-80	Beta-1,3-endoglucanase, putative pathogenesis-related protein 2 (PR-2)	defense response to fungus, incompatible interaction (GO:0009817); multi-organism process (GO:0051704); innate immune response (GO:0045087); response to stimulus (GO:0050896); immune response (GO:0006955); organic substance metabolic process (GO:0071704); immune system process (GO:0002376); metabolic process (GO:0008152); response to external biotic stimulus (GO:0043207); response to other organism (GO:0051707); response to stress (GO:0006950); response to biotic stimulus (GO:0009607); primary metabolic process (GO:0044238); response to salt stress (GO:0009651); apoplast (GO:0048046); catalytic activity (GO:0003824)	1.05	2.87E-07	0.000671
CR393796	PITA_000018776-RA	AT2G24210	233	6e-61	TERPENE SYNTHASE 10 (TPS10)	response to organic substance (GO:0010033); single organism reproductive process (GO:0044702); multi-organism process (GO:0051704); response to stimulus (GO:0050896); developmental process involved in reproduction (GO:0003006); organic substance metabolic process (GO:0071704); single-organism cellular process (GO:0044763); response to hormone (GO:0009725); reproductive process (GO:0022414); negative regulation of biological process (GO:0048519); regulation of response to stress (GO:0080134); metabolic process (GO:0008152); gametophyte development (GO:0048229); jasmonic acid mediated signaling pathway (GO:0009867); cellular response to jasmonic acid stimulus	1.05	0.034771	0.505357

						(GO:0071395); single-organism process (GO:0044699); reproductive structure development (GO:0048608); reproductive system development GO:0061458); response to bacterium (GO:0009617); response to oxygen-containing compound (GO:1901700); anatomical structure development (GO:0048856); response to endogenous stimulus (GO:0009719); single-organism developmental process (GO:0044767); post-embryonic development (GO:0009791); multicellular organismal development (GO:0007275); response to external biotic stimulus (GO:0043207); response to other organism (GO:0051707); regulation of defense response (GO:0031347); response to stress (GO:0006950); developmental process (GO:0032502); response to jasmonic acid (GO:0009753); response to chemical (GO:0042221); single-multicellular organism process (GO:0044707); response to biotic stimulus (GO:0009607); primary metabolic process (GO:0044238); cellular response to stimulus (GO:0051716); regulation of response to stimulus (GO:0048583); multicellular organismal process (GO:0032501); response to acid chemical (GO:0001101)			
BX255736	PITA_000067285-RA	AT5G10260	372	1e-103	RAB GTPase homolog H1E (RABH1e)	response to stimulus (GO:0050896); single-organism cellular process GO:0044763); single-organism process (GO:0044699); cellular response to stimulus (GO:0051716); carbohydrate derivative binding (GO:0097367); purine ribonucleoside triphosphate binding	1.02	0.000379	0.054605

						(GO:0035639); purine nucleoside binding (GO:0001883); purine ribonucleoside binding (GO:0032550); ribonucleoside binding (GO:0032549); nucleoside binding (GO:0001882); purine ribonucleotide binding (GO:0032555); purine nucleotide binding (GO:0017076); nucleotide binding (GO:0000166); nucleoside phosphate binding (GO:1901265); ribonucleotide binding (GO:0032553); anion binding (GO:0043168); small molecule binding (GO:0036094)			
BX249322	PITA_000085327-RA	AT5G23860	117	2e-27	Beta-tubulin 8 (TUB8)	single organism reproductive process (GO:0044702); tissue development (GO:0009888); response to stimulus (GO:0050896); developmental process involved in reproduction (GO:0003006); single-organism cellular process (GO:0044763); reproductive process (GO:0022414); cellular process involved in reproduction in multicellular organism (GO:0022412); seed development (GO:0048316); (GO:0008152); single-organism process (GO:0044699); reproductive structure development (GO:0048608); reproductive system development (GO:0061458); fruit development (GO:0010154): anatomical structure development (GO:0048856); multicellular organismal development (GO:0007275); multicellular organism reproduction (GO:0032504); seed trichome elongation (GO:0090378); single-organism developmental process (GO:0044767); post-embryonic development (GO:0009791); multicellular organismal development (GO:0007275);	1.00	0.008408	0.295989

						response to stress (GO:0006950); developmental process (GO:0032502); single-multicellular organism process (GO:0044707); seed trichome differentiation (GO:0090376); response to salt stress (GO:0009651); multicellular organismal process (GO:0032501); plant epidermis development (GO:0090558); catalytic activity (GO:0003824); carbohydrate derivative binding (GO:0097367); purine ribonucleoside triphosphate binding (GO:0035639); purine nucleoside binding (GO:0001883); purine ribonucleoside binding (GO:0032550); ribonucleoside binding (GO:0032549); nucleoside binding (GO:0001882); purine ribonucleotide binding (GO:0032555); purine nucleotide binding (GO:0017076); nucleotide binding (GO:0000166); nucleoside phosphate binding (GO:1901265); ribonucleotide binding (GO:0032553); anion binding (GO:0043168); small molecule binding (GO:0036094)			
BX249246	PITA_000050649-RA	AT5G05390	717	0.0	Putative Laccase 12 (LAC12), laccase family protein	organic substance metabolic process (GO:0071704); single-organism cellular process (GO:0044763); metabolic process GO:0008152); single-organism process (GO:0044699); organic substance catabolic process (GO:1901575); single-organism metabolic process (GO:0044710); apoplast (GO:0048046); catalytic activity (GO:0003824)	0.99	0.028193	0.475341
BX680644	PITA_000068951-RA	AT1G20160	144	1e-34	CO2 Response Secreted Protease (CRSP), cell wall	response to organic substance (GO:0010033); tissue development (GO:0009888); response to stimulus (GO:0050896); organic substance	0.97	0.025418	0.451252

					localized subtilisin-like serine protease	metabolic process (GO:0071704); negative regulation of biological process (GO:0048519); metabolic process (GO:0008152); single-organism process (GO:0044699); response to oxygen-containing compound (GO:1901700); anatomical structure development (GO:0048856); negative regulation of post-embryonic development (GO:0048581); single-organism developmental process (GO:0044767); post-embryonic development (GO:0009791); multicellular organismal development (GO:0007275); developmental process (GO:0032502); response to chemical (GO:0042221); single-multicellular organism process (GO:0044707); negative regulation of multicellular organismal process (GO:0051241); primary metabolic process (GO:0044238); negative regulation of developmental process (GO:0051093); multicellular organismal process (GO:0032501); plant epidermis development (GO:0090558); apoplast (GO:0048046); catalytic activity (GO:0003824)			
BX254429	PITA_000026838-RA	AT4G02570	434	1e-122	CULLIN 1 (CUL1), component of SCF ubiquitin ligase complex	response to organic substance (GO:0010033); single organism reproductive process (GO:0044702); tissue development (GO:0009888); response to stimulus (GO:0050896); developmental process involved in reproduction (GO:0003006); organic substance metabolic process (GO:0071704); single-organism cellular process (GO:0044763); response to hormone (GO:0009725); reproductive process (GO:0022414); seed	0.97	0.013609	0.351883

						development (GO:0048316); metabolic process (GO:0008152); jasmonic acid mediated signaling pathway (GO:0009867); cellular response to jasmonic acid stimulus (GO:0071395); single-organism process (GO:0044699); reproductive structure development (GO:0048608); reproductive system development GO:0061458); response to oxygen-containing compound (GO:1901700); fruit development (GO:0010154); anatomical structure development (GO:0048856); response to endogenous stimulus (GO:0009719); ethylene-activated signaling pathway (GO:0009873); response to ethylene (GO:0009723 single-organism developmental process (GO:0044767); post-embryonic development (GO:0009791); cellular response to ethylene stimulus (GO:0071369); multicellular organismal development (GO:0007275); developmental process (GO:0032502); response to jasmonic acid (GO:0009753); response to chemical (GO:0042221); single-multicellular organism process (GO:0044707); embryo development ending in seed dormancy (GO:0009793); primary metabolic process (GO:0044238); phosphorelay signal transduction system (GO:0000160); embryo development (GO:0009790); organic substance catabolic process (GO:1901575); cellular response to stimulus (GO:0051716); multicellular organismal process (GO:0032501); response to acid chemical			

						(GO:0001101); catalytic activity (GO:0003824)			
CR393151	PITA_000076085-RA	AT5G23680	32	0.34	Long chain base (LCB) sphingolipid intermediate LCB2a	-	0.95	1.04E-05	0.005589
BX682957	PITA_000032031-RA	AT3G18110	167	5e-42	Embryo Defective 1270 (EMB1270), Pentatricopeptide repeat family protein	single organism reproductive process (GO:0044702); developmental process involved in reproduction (GO:0003006); reproductive process (GO:0022414); seed development (GO:0048316); single-organism process (GO:0044699); reproductive structure development (GO:0048608); reproductive system development (GO:0061458); fruit development (GO:0010154); anatomical structure development (GO:0048856); single-organism developmental process (GO:0044767); post-embryonic development (GO:0009791); multicellular organismal development (GO:0007275); developmental process (GO:0032502); single-multicellular organism process (GO:0044707); embryo development ending in seed dormancy (GO:0009793); embryo development (GO:0009790); multicellular organismal process (GO:0032501)	0.94	0.018598	0.408968
BX681904	PITA_000038025-RA	AT5G03840	197	3e-51	TERMINAL FLOWER 1 (TFL1), phosphatidylethanolamine-binding protein	response to organic substance (GO:0010033); single organism reproductive process (GO:0044702); response to stimulus (GO:0050896); developmental process involved in reproduction (GO:0003006); organic substance metabolic process (GO:0071704); response to sucrose (GO:0009744); response to disaccharide (GO:0034285); single-organism cellular process (GO:0044763); reproductive	0.92	0.035358	0.505357

						process (GO:0022414); negative regulation of biological process (GO:0048519); metabolic process (GO:0008152); single-organism process (GO:0044699); reproductive structure development (GO:0048608); reproductive system development (GO:0061458); response to oxygen-containing compound (GO:1901700); anatomical structure development (GO:0048856): negative regulation of post-embryonic development (GO:0048581); single-organism developmental process (GO:0044767); post-embryonic development (GO:0009791); multicellular organismal development (GO:0007275); developmental process (GO:0032502); response to chemical (GO:0042221); single-multicellular organism process (GO:0044707); negative regulation of multicellular organismal process (GO:0051241); primary metabolic process (GO:0044238); negative regulation of developmental process (GO:0051093); response to carbohydrate (GO:0009743); multicellular organismal process (GO:0032501); negative regulation of flower development (GO:0009910); anion binding (GO:0043168)			
BX255023	PITA_000021699-RA	AT1G03520	290	1e-78	Core-2/I-branching beta-1,6-N-acetylglucosaminyltransferase family protein	organic substance metabolic process (GO:0071704); metabolic process (GO:0008152); single-organism process (GO:0044699); primary metabolic process (GO:0044238); single-organism metabolic process (GO:0044710); catalytic activity (GO:0003824)	0.92	0.011935	0.328637

BX681045	PITA_000077629-RA	AT3G57450	62	1e-10	Unknown protein T8H10.50	-	0.89	0.002732	0.172418
BX680490	PITA_000083084-RA	AT3G24240	1101	0.0	Leucine-rich receptor-like protein kinase family protein RFGR	multi-organism process (GO:0051704); tissue development (GO:0009888); response to stimulus (GO:0050896); organic substance metabolic process (GO:0071704); single-organism cellular process (GO:0044763); metabolic process (GO:0008152); single-organism process (GO:0044699); multi-organism cellular process (GO:0044764); anatomical structure development (GO:0048856); symbiosis, encompassing mutualism through parasitism (GO:0044403); interspecies interaction between organisms (GO:0044419); single-organism developmental process (GO:0044767); multicellular organismal development (GO:0007275); developmental process (GO:0032502); single-multicellular organism process (GO:0044707); primary metabolic process (GO:0044238); cellular response to stimulus (GO:0051716); transmembrane receptor protein tyrosine kinase signaling pathway (GO:0007169); multicellular organismal process (GO:0032501); enzyme linked receptor protein signaling pathway (GO:0007167); catalytic activity (GO:0003824); response to acid chemical (GO:0001101); single-organism metabolic process (GO:0044710); catalytic activity (GO:0003824); carbohydrate derivative binding (GO:0097367); purine ribonucleoside triphosphate binding (GO:0035639); purine nucleoside binding (GO:0001883); purine	0.88	0.00930291	0.303587

						ribonucleoside binding (GO:0032550); ribonucleoside binding (GO:0032549); nucleoside binding (GO:0001882); purine ribonucleotide binding (GO:0032555); purine nucleotide binding (GO:0017076); nucleotide binding (GO:0000166); nucleoside phosphate binding (GO:1901265); ribonucleotide binding (GO:0032553); ATP binding (GO:0005524); anion binding (GO:0043168); small molecule binding (GO:0036094); adenyl ribonucleotide binding (GO:0032559); adenyl nucleotide binding (GO:0030554); protein serine/threonine kinase activity (GO:0004674)			
BX253270	PITA_000039871-RA	AT3G49210	65	3e-11	O-fucosyltransferase family protein	organic substance metabolic process (GO:0071704); single-organism cellular process (GO:0044763); metabolic process (GO:0008152); single-organism process (GO:0044699); primary metabolic process (GO:0044238); single-organism metabolic process (GO:0044710); catalytic activity (GO:0003824)	0.87	0.016348	0.388875
BX678105	PITA_000030236-RA	AT1G14520	445	1e-125	Myo-Inositol Oxygenase 1 (MIOX1), myo-inositol oxygenase gene family	response to organic substance (GO:0010033); single organism reproductive process (GO:0044702); metabolic process (GO:0008152); single-organism process (GO:0044699); anatomical structure development (GO:0048856); single-organism developmental process (GO:0044767); developmental process (GO:0032502); primary metabolic process (GO:0044238); organic substance catabolic process (GO:1901575); monosaccharide biosynthetic process (GO:0046364); single-organism	0.81	0.000458	0.061297

						metabolic process (GO:0044710); catalytic activity (GO:0003824)			
BX676888	PITA_000051106-RA	AT1G08080	257	2e-68	Alpha Carbonic Anhydrase 7 (ACA7)	single-organism cellular process (GO:0044763); metabolic process (GO:0008152); single-organism process (GO:0044699); single-organism metabolic process (GO:0044710); catalytic activity (GO:0003824)	1.88	0.016509	0.39117
BX679533	PITA_000007770-RA	AT3G52950	609	1e-174	CBS / octicosapeptide/Phox/Bemp1 (PB1) domains-containing protein	-	0.76	0.006837	0.270915
BX678533	PITA_000002174-RA	AT1G43760	63	7e-10	DNAse I-like superfamily protein	-	0.76	0.008081	0.292513
BX678097	PITA_000014236-RA	AT4G36040	90	2e-18	DNA J PROTEIN C23 (DJC23), chaperone DnaJ-domain superfamily protein	organic substance metabolic process (GO:0071704); metabolic process (GO:0008152); primary metabolic process (GO:0044238)	0.69	0.024127	0.437998
BX682473	PITA_000005269-RA	AT3G23150	396	1e-110	ETHYLENE RESPONSE 2 (ETR2), ethylene receptor, subfamily 2	response to organic substance (GO:0010033); response to stimulus GO:0050896); organic substance metabolic process GO:0071704); negative regulation of ethylene-activated signaling pathway (GO:0010105); negative regulation of phosphorelay signal transduction system (GO:0070298); single-organism cellular process (GO:0044763); response to hormone (GO:0009725); negative regulation of biological process (GO:0048519); metabolic process (GO:0008152); regulation of ethylene-activated signaling pathway (GO:0010104); regulation of phosphorelay signal transduction system (GO:0070297); negative regulation of intracellular signal transduction (GO:1902532); single-organism process	0.69	0.005514	0.239775

						(GO:0044699); response to endogenous stimulus (GO:0009719); ethylene-activated signaling pathway (GO:0009873); response to ethylene (GO:0009723); cellular response to ethylene stimulus (GO:0071369); response to chemical (GO:0042221); primary metabolic process (GO:0044238); phosphorelay signal transduction system (GO:0000160); cellular response to stimulus (GO:0051716); regulation of response to stimulus (GO:0048583); single-organism metabolic process (GO:0044710); catalytic activity (GO:0003824); carbohydrate derivative binding (GO:0097367); purine ribonucleoside triphosphate binding (GO:0035639); purine nucleoside binding (GO:0001883); purine ribonucleoside binding (GO:0032550); ribonucleoside binding (GO:0032549); nucleoside binding (GO:0001882); purine ribonucleotide binding (GO:0032555); purine nucleotide binding (GO:0017076); nucleotide binding (GO:0000166); nucleoside phosphate binding (GO:1901265); ribonucleotide binding (GO:0032553); ATP binding (GO:0005524); anion binding (GO:0043168); small molecule binding (GO:0036094); adenyl ribonucleotide binding (GO:0032559); adenyl nucleotide binding (GO:0030554); protein serine/threonine kinase activity (GO:0004674)			
BX252125	PITA_000041961-RA	AT4G33300	483	1e-136	ACTIVATED DISEASE RESISTANCE-LIKE 1	multi-organism process (GO:0051704); response to stimulus	0.68	0.010057	0.310496

					(ADR1-L1), ADR1 family nucleotide-binding leucine-rich repeat (NB-LRR) immune receptor	(GO:0050896); response to bacterium (GO:0009617); response to external biotic stimulus (GO:0043207); response to other organism (GO:0051707); response to stress (GO:0006950); response to biotic stimulus (GO:0009607); apoplast (GO:0048046); carbohydrate derivative binding (GO:0097367); purine ribonucleoside triphosphate binding (GO:0035639); purine nucleoside binding (GO:0001883); purine ribonucleoside binding (GO:0032550); ribonucleoside binding (GO:0032549); nucleoside binding (GO:0001882); purine ribonucleotide binding (GO:0032555); purine nucleotide binding (GO:0017076); nucleotide binding (GO:0000166); nucleoside phosphate binding (GO:1901265); ribonucleotide binding (GO:0032553); ATP binding (GO:0005524); anion binding (GO:0043168); small molecule binding (GO:0036094); adenyl ribonucleotide binding (GO:0032559); adenyl nucleotide binding (GO:0030554)			
BX680472	PITA_000004496-RA	AT5G02860	815	0.0	Pentatricopeptide repeat (PPR) superfamily protein	-	0.68	0.032873	0.497032
BX251316	PITA_000055597-RA	AT1G03090	92	8e-20	MCCA, methylcrotonoyl-CoA carboxylase non-biotinylated subunit protein	organic substance metabolic process (GO:0071704); single-organism cellular process (GO:0044763); metabolic process (GO:0008152); single-organism process (GO:0044699); primary metabolic process (GO:0044238); organic substance catabolic process (GO:1901575); single-organism metabolic process (GO:0044710); catalytic activity	0.68	0.03316	0.497643

						(GO:0003824); carbohydrate derivative binding (GO:0097367); purine ribonucleoside triphosphate binding (GO:0035639); purine nucleoside binding (GO:0001883); purine ribonucleoside binding (GO:0032550); ribonucleoside binding (GO:0032549); nucleoside binding (GO:0001882); purine ribonucleotide binding (GO:0032555); purine nucleotide binding (GO:0017076); nucleotide binding (GO:0000166); nucleoside phosphate binding (GO:1901265); ribonucleotide binding (GO:0032553); ATP binding (GO:0005524); anion binding (GO:0043168); small molecule binding (GO:0036094); adenyl ribonucleotide binding (GO:0032559); adenyl nucleotide binding (GO:0030554); protein serine/threonine kinase activity (GO:0004674)			
BX254280	PITA_000040562-RA	AT5G26680	248	33e-66	5'-3' exonuclease family protein	response to stimulus (GO:0050896); organic substance metabolic process (GO:0071704); single-organism cellular process (GO:0044763); metabolic process (GO:0008152); single-organism process (GO:0044699); response to stress (GO:0006950); primary metabolic process (GO:0044238); organic substance catabolic process (GO:1901575); cellular response to stimulus (GO:0051716); single-organism metabolic process (GO:0044710); catalytic activity (GO:0003824)	0.67	0.034905	0.505357
CR393785	PITA_000094396-RA	AT1G72060	62	2e-10	Serine-type endopeptidase inhibitor	response to stimulus (GO:0050896); organic substance metabolic process (GO:0071704); negative regulation of biological process (GO:0048519); metabolic process	0.65	0.00015	0.034101

						(GO:0008152); response to stress (GO:0006950): primary metabolic process (GO:0044238)			
BX250402	PITA_00001891 4-RA	AT2G36310	218	5e-57	NUCLEOSIDE HYDROLASE 1 (NSH1)	organic substance metabolic process (GO:0071704); single-organism cellular process (GO:0044763); metabolic process (GO:0008152); single-organism process (GO:0044699); primary metabolic process (GO:0044238); organic substance catabolic process (GO:1901575); single-organism metabolic process (GO:0044710); catalytic activity (GO:0003824)	0.65	0.008957	0.299909
BX253641	PITA_00002507 7-RA	AT5G25190	137	2e-32	ETHYLENE RESPONSIVE TRANSCRIPTION FACTOR 3 (ERF003), member of subfamily B-6 of ERF/AP2 transcription factor family	response to organic substance (GO:0010033); response to stimulus (GO:0050896); organic substance metabolic process (GO:0071704); single-organism cellular process (GO:0044763); response to hormone (GO:0009725); metabolic process (GO:0008152); single-organism process (GO:0044699); response to endogenous stimulus (GO:0009719); ethylene-activated signaling pathway (GO:0009873); response to ethylene (GO:0009723); cellular response to ethylene stimulus (GO:0071369); response to chemical (GO:0042221); primary metabolic process (GO:0044238); phosphorelay signal transduction system (GO:0000160); cellular response to stimulus (GO:0051716)	0.64	0.027855	0.470761
BX681974	PITA_00001534 0-RA	AT5G42300	148	7e-37	UBIQUITIN-LIKE PROTEIN 5 (UBL5), ubiquitin-like superfamily protein	organic substance metabolic process (GO:0071704); metabolic process (GO:0008152); primary metabolic process (GO:0044238); (GO:0032553); ATP binding (GO:0005524); anion binding (GO:0043168); small molecule binding (GO:0036094); adenyl	0.63	0.012536	0.339955

						ribonucleotide binding (GO:0032559); adenyl nucleotide binding (GO:0030554)			
BX680855	PITA_000004184-RA	AT1G77120	546	1e-155	Alcohol Dehydrogenase 1 (ADH1)	response to organic substance (GO:0010033); response to stimulus (GO:0050896); response to sucrose (GO:0009744); response to disaccharide (GO:0034285); single-organism cellular process (GO:0044763); response to hormone (GO:0050896); regulation of response to stress (GO:0080134); metabolic process (GO:0008152); single-organism process (GO:0044699); response to oxygen-containing compound (GO:1901700); response to endogenous stimulus (GO:0009719); response to stress (GO:0006950); response to chemical (GO:0042221); response to salt stress (GO:0009651); cellular response to stimulus (GO:0051716); response to carbohydrate (GO:0009743); regulation of response to stimulus (GO:0048583); response to acid chemical (GO:0001101); single-organism metabolic process (GO:0044710); catalytic activity (GO:0003824); nucleotide binding (GO:0000166); nucleoside phosphate binding (GO:1901265); small molecule binding (GO:0036094)	0.63	0.017152	0.396094
BX682159	PITA_000050150-RA	AT5G07990	377	1e-104	Cytochrome P450 75B1 (CYP75B1)	response to organic substance (GO:0010033); response to stimulus (GO:0050896); organic substance metabolic process (GO:0071704); response to hormone (GO:0050896); metabolic process (GO:0008152); single-organism process (GO:0044699); response to hormone (GO:0009725); response to endogenous stimulus	0.61	0.005921	0.248423

						(GO:0009719); response to chemical (GO:0042221); single-organism metabolic process (GO:0044710); catalytic activity (GO:0003824)			
BX677372	PITA_000047909-RA	AT3G60720	84	2e-16	Plasmodesmata-Located Protein 8 (PDLP8)	multi-organism process (GO:0051704); multi-organism cellular process (GO:0044764); symbiosis, encompassing mutualism through parasitism (GO:0044403); interspecies interaction between organisms (GO:0044419)	0.60	0.030528	0.4849
BX680146	PITA_000035441-RA	AT3G60720	64	3e-11	Plasmodesmata-Located Protein 8 (PDLP8)	multi-organism process (GO:0051704); multi-organism cellular process (GO:0044764); symbiosis, encompassing mutualism through parasitism (GO:0044403); interspecies interaction between organisms (GO:0044419)	0.60	0.011637	0.326025
BX680264	PITA_000032339-RA	AT5G03690	175	2e-52	FBA4 Aldolase superfamily protein	nucleus (GO:0005730); cytoplasm(GO:0005737); mitochondrion (GO:0005739); mitochondrial envelope (GO:0005740); plasma membrane (GO:0005886); chloroplast (GO:0009507); apoplast (GO:0048046); cell wall (GO:0005618); fructose-bisphosphate aldolase activity (GO:0004332); protein binding (GO:0005515); glycolytic process (GO:0006096); response to hypoxia (GO:0001666); response to salt stress (GO:0009651); response to cadmium ion (GO:0046686)	0.59	0.003268	0.182065
BX677882	PITA_000083353-RA	AT3G02940	211	1e-54	MYB DOMAIN PROTEIN 74 (MYB74)	response to organic substance (GO:0010033); response to stimulus (GO:0050896); organic substance metabolic process (GO:0071704); single-organism cellular process (GO:0044763); metabolic process (GO:0008152); single-organism process (GO:0044699);	0.58	0.016106	0.388875

						response to oxygen-containing compound (GO:1901700); single-organism developmental process (GO:0044767); developmental process (GO:0032502); response to chemical (GO:0042221); primary metabolic process (GO:0044238); response to acid chemical (GO:0001101)			
BX676894	PITA_000036738-RA	AT4G24660	164	2e-49	ATHB22, MEE68, HB22, ZHD2 homeobox protein 22	-	0.56	0.000902	0.093424
BX677095	PITA_000005919-RA	AT4G33510	750	0.0	3-DEOXY-D-ARABINO-HEPTULOSONATE-7-PHOSPHATE 2 (DAHP2), Class-II DAHP synthetase family protein	organic substance metabolic process (GO:0071704); single-organism cellular process (GO:0044763); metabolic process (GO:0008152); single-organism process (GO:0044699); primary metabolic process (GO:0044238); single-organism metabolic process (GO:0044710); catalytic activity (GO:0003824)	0.56	0.006656	0.266706
BX679807	PITA_000003863-RA	AT1G56430	292	4e-79	NICOTIANAMINE SYNTHASE 4 (NAS4)	single organism reproductive process (GO:0044702); multi-organism process (GO:0051704); developmental process involved in reproduction (GO:0003006); organic substance metabolic process (GO:0071704); single-organism cellular process (GO:0044763); reproductive process (GO:0022414); metabolic process (GO:0008152); gametophyte development (GO:0048229); single-organism process (GO:0044699); anatomical structure development (GO:0048856); single-organism developmental process (GO:0044767); multicellular organismal development (GO:0007275); developmental process (GO:0032502); single-multicellular organism process (GO:0044707);	0.56	0.038497	0.530294

						multicellular organismal process (GO:0032501); single-organism metabolic process (GO:0044710); catalytic activity (GO:0003824)			
BX250160	PITA_000038492-RA	AT1G74670	130	8e-31	GA-STIMULATED ARABIDOPSIS 6 (GASA6), Gibberellin-regulated family protein	response to organic substance (GO:0010033); response to stimulus (GO:0050896); response to sucrose (GO:0009744); response to disaccharide (GO:0034285); single-organism cellular process (GO:0044763); response to hormone (GO:0009725); single-organism process (GO:0044699); response to oxygen-containing compound (GO:1901700); response to endogenous stimulus (GO:0009719); response to chemical (GO:0042221); cellular response to stimulus (GO:0051716); response to carbohydrate (GO:0009743); response to acid chemical (GO:0001101)	0.56	0.001434	0.117623
BX250343	PITA_000004398-RA	AT1G71050	174	2e-56	Heavy metal transport/detoxification superfamily protein	plasma membrane (GO:0005886); metal ion binding (GO:0046872); metal ion transport (GO:0030001)	0.54	0.021453	0.425013
BX253073	PITA_000065499-RA	AT3G58970	86	2e-17	MAGNESIUM TRANSPORTER 6 (MGT6), transmembrane magnesium transporter	single-organism cellular process (GO:0044763); single-organism process (GO:0044699)	0.53	0.008329	0.295548
BX784340	PITA_000041017-RA	AT4G35530	40	1e-05	Phosphatidylinositol N-acetylglucosaminyltransferase	-	0.51	0.024338	0.439431
BX254612	PITA_000087331-RA	AT1G07400	161	4e-51	HSP20-like chaperones superfamily protein	-	0.50	0.016717	0.392682

CR394028	PITA_000052636-RA	AT3G53260	995	0	PAL2, ATPAL2 phenylalanine ammonia-lyase 2	cytoplasm (GO:0005737); ammonia ligase activity (GO:0016211); yase activity (GO:0016829); ammonia-lyase activity (GO:0016841); phenylalanine ammonia-lyase activity (GO:0045548); biosynthetic process (GO:0009058); phenylpropanoid metabolic process (GO:0009698); L-phenylalanine catabolic process (GO:0006559)	0.49	0.012797	0.344418
BX251802	PITA_000036476-RA	AT3G07790	429	6e-146	DGCR14-related	-	0.48	0.002231	0.153003
AL749806	PITA_000064132-RA	AT5G56000	377	2e-128	Hsp81.4, AtHsp90.4 HEAT SHOCK PROTEIN 81.4	ATP binding (GO:0005524); unfolded protein binding (GO:0051082); protein folding (GO:0006457); response to stress (GO:0006950)	0.48	0.004547	0.215119
AL751228	PITA_000010369-RA	AT1G23120	40	0.0000438	Polyketide cyclase/dehydrase and lipid transport superfamily protein	defense response (GO:0006952); response to biotic stimulus (GO:0009607)	0.47	1.06268E-05	0.005589
CR354759	PITA_000058715-RA	AT5G62000	280	5e-89	Auxin response factor 2	plastid (GO:0009536); nucleus (GO:0005634); DNA binding (GO:0003677); DNA-binding transcription factor activity (GO:0003700); protein binding (GO:0005515); obsolete transcription regulator activity (GO:0030528); double fertilization forming a zygote and endosperm (GO:0009567); red, far-red light phototransduction (GO:0009585); de-etiolation (GO:0009704); response to cytokinin (GO:0009735); esponse to gibberellin (GO:0009739); positive regulation of flower development (GO:0009911); red light signaling pathway (GO:0010161); regulation of transcription (GO:0006355); cellular protein modification process (GO:0006464); multicellular organism development	0.47	2.34986E-05	0.00931

						(GO:0007275); petal morphogenesis (GO:0048446)			
**Down-Regulated Genes**									
BX253783	PITA_000011239-RA	AT5G56130	260	1e-95	Putative TEX1, component of THO/TREX mRNA export complex	transport (GO:0006810); cellular nitrogen compound metabolic process (GO:0034641); mRNA processing (GO:0006397)	-2.05	0.024025	0.437998
BX680222	PITA_000026854-RA	AT4G25340	32	0.40	FK506 BINDING PROTEIN 53 (FKBP53), member of the FKBP-type immunophilin family	cellular protein modification process (GO:0006464); cellular component assembly (GO:0022607); protein folding (GO:0006457); macromolecular complex assembly (GO:0065003); protein complex assembly (GO:0006461); chromosome organization (GO:0051276);	-1.23	0.000185	0.035526
BX250930	PITA_000058370-RA	AT4G03210	333	1e-91	XYLOGLUCAN ENDOTRANSGLUCOSYLASE/HYDROLASE 9 (XTH9)	anatomical structure development (GO:0048856); growth (GO:0040007); carbohydrate metabolic process (GO:0005975)	-1.01	0.010482	0.317464
AL749574	PITA_000012986-RA	AT5G06320	112	4e-25	NDR1/HIN1-LIKE 3 (NHL3)	response to stress (GO:0006950); immune system process (GO:0002376)	-1.01	0.019106	0.413665
AL750772	PITA_000044560-RA	AT3G56440	122	2e-28	HOMOLOG OF YEAST AUTOPHAGY 18 (ATG18) D	biosynthetic process (GO:0009058); response to stress (GO:0006950); catabolic process (GO:0009056); cellular protein modification process (GO:0006464); cellular component assembly (GO:0022607); autophagy (GO:0006914); mitochondrion organization (GO:0007005)	-0.99	0.022642	0.429477
CR392068	PITA_000065501-RA	AT3G54720	579	1e-165	ALTERED MERISTEM PROGRAM 1 (AMP1), peptidase M28 family protein	-	-0.98	0.003456	0.186124
CR394334	PITA_000088914-RA	AT2G16800	30	0.61	High-affinity nickel-transport family protein	biosynthetic process (GO:0009058); response to stress (GO:0006950); transport (GO:0006810); cellular nitrogen compound metabolic process	-0.96	0.000632	0.075125

						(GO:0034641); small molecule metabolic process (GO:0044281); immune system process (GO:0002376); transmembrane transport (GO:0055085); cellular amino acid metabolic process (GO:0006520); symbiosis, encompassing mutualism through parasitism (GO:0044403); sulfur compound metabolic process (GO:0006790)			
BX680630	PITA_000003863-RA	AT1G09240	293	3e-79	NICOTIANAMINE SYNTHASE 3 (NAS3)	transport (GO:0006810); anatomical structure development (GO:0048856); cellular nitrogen compound metabolic process (GO:0034641); small molecule metabolic process (GO:0044281); growth (GO:0040007); cell differentiation (GO:0030154); cell morphogenesis (GO:0000902)	-0.96	0.004653	0.216875
BX677134	PITA_000044002-RA	AT4G14746	95	1e-19	Neurogenic locus notch-like protein	-	-0.91	4.7E-06	0.004868
CR392755	PITA_000093728-RA	AT2G32690	61	2e-09	ATGRP23, GRP23 | glycine-rich protein	-	-0.87	8.19E-05	0.020858
BX254845	PITA_000000579-RA	AT5G65000	379	1e-105	Nucleotide-sugar transporter family protein ROCK1	catabolic process (GO:0009056); cell wall organization or biogenesis (GO:0071554); cellular component assembly (GO:0022607); transmembrane transport (GO:0055085); anatomical structure formation involved in morphogenesis (GO:0048646); extracellular matrix organization (GO:0030198)	-0.85	0.000237	0.04153
CR394036	PITA_000033329-RA	AT3G16440	68	3e-14	Jacalin-related lectin 32	intrinsic component of membrane (GO:0031224); transmembrane signaling receptor activity (GO:0004888); signal	-0.81	0.04291	0.549758

						transduction (GO:0007165); innate immune response (GO:0045087);			
BX678830	PITA_000025958-RA	AT3G10260	246	3e-6	Reticulon family protein	biosynthetic process (GO:0009058); response to stress (GO:0006950); transport (GO:0006810); immune system process (GO:0002376); protein targeting (GO:0006605); cell death (GO:0008219); membrane organization (GO:0061024)	-0.79	0.010915	0.322371
BX784084	PITA_000055747-RA	AT4G34580	241	2e-63	Sec14p-like phosphatidylinositol transfer family protein (COW1)	growth (GO:0040007); cell differentiation (GO:0030154); cell morphogenesis (GO:0000902); developmental maturation (GO:0021700)	-0.77	0.010513	0.317464
BX251115	PITA_000025277-RA	AT1G10650	243	4e-64	SBP (S-ribonuclease binding protein) family protein	-	-0.76	0.046763	0.569142
CR392761	PITA_000058463-RA	AT5G43860	238	7e-63	ATCLH2, CLH2 | chlorophyllase 2	cellular nitrogen compound metabolic process (GO:0034641); catabolic process (GO:0009056); cofactor metabolic process (GO:0051186)	-0.76	5.03E-07	0.000717
BX250940	PITA_000004343-RA	AT2G42780	65	2e-10	transcription elongation factor B polypeptide	biosynthetic process (GO:0009058); cellular nitrogen compound metabolic process (GO:0034641)	-0.76	0.002432	0.163618
BX679601	PITA_000006323-RA	AT5G41040	580	0	HXXXD-type acyl-transferase family protein	-	-0.76	0.047830	0.570158
BX252702	PITA_000026873-RA	AT1G12280	178	3e-44	putative NBS/LRR disease resistance protein SUMM2	response to stress (GO:0006950)	-0.75	0.016347	0.388875
BX678708	PITA_000027212-RA	AT2G02850	110	8e-25	ARPN | plantacyanin	response to stress (GO:0006950); anatomical structure development (GO:0048856); signal transduction (GO:0007165); cellular protein modification process (GO:0006464); reproduction (GO:0000003); embryo development (GO:0009790)	-0.75	4.95E-06	0.004868

BX679532	PITA_000008136-RA	AT5G67070	87	4e-18	ralf-like 34 (RALFL34)	anatomical structure development (GO:0048856); anatomical structure formation involved in morphogenesis (GO:0048646); cell-cell signaling (GO:0007267)	-0.71	0.017158	0.396094
AL749765	PITA_000073027-RA	AT3G18810	37	0.009	Protein kinase superfamily protein (PERK6)	anatomical structure development (GO:0048856); signal transduction (GO:0007165); growth (GO:0040007); cellular protein modification process (GO:0006464); cell morphogenesis (GO:0000902)	-0.69	1.47E-05	0.006534
AL750172	PITA_000007900-RA	AT5G60910	60	2e-09	AGL8, FUL | AGAMOUS-like 8	biosynthetic process (GO:0009058); anatomical structure development (GO:0048856); cellular nitrogen compound metabolic process (GO:0034641); cell differentiation (GO:0030154)	-0.69	0.020445	0.419482
BX677880	PITA_000029613-RA	AT5G03680	240	3e-63	Duplicated homeodomain-like superfamily protein (PTL)	biosynthetic process (GO:0009058); anatomical structure development (GO:0048856); cellular nitrogen compound metabolic process (GO:0034641); growth (GO:0040007); anatomical structure formation involved in morphogenesis (GO:0048646)	-0.68	0.000464	0.061297
CR394371	PITA_000047990-RA	AT5G67190	139	7e-40	Ethylene-responsive transcription factor ERF010 / DEAR2	nucleus (GO:0005634); zinc ion binding (GO:0008270); DNA-binding transcription factor activity (GO:0003700); plant-type secondary cell wall biogenesis (GO:0009834); ethylene-activated signaling pathway (GO:0009873); response to chitin (GO:0010200); regulation of transcription, DNA-templated (GO:0006355)	-0.66	0.031959	0.491224
BX250614	PITA_000028580-RA	AT5G51550	413	1e-115	EXORDIUM like 3 (EXL3)	cellular component assembly (GO:0022607); macromolecular complex assembly (GO:0065003);	-0.65	3.76E-07	0.000671

						protein complex assembly (GO:0006461); cytoskeleton organization (GO:0007010)			
BX681731	PITA_000007482-RA	AT5G15780	83	3e-16	Pollen Ole e 1 allergen and extensin family protein	-	-0.65	0.00128	0.112933
BX253560	PITA_000016812-RA	AT3G23770	427	1e-146	O-Glycosyl hydrolases family 17 protein	anchored component of plasma membrane (GO:0046658); extracellular region (GO:0005576); cell wall (GO:0005618); plant-type cell wall (GO:0009505); anchored component of membrane (GO:0031225); glucan endo-1,3-beta-D-glucosidase activity (GO:0042973); cation binding (GO:0043169); hydrolase activity, hydrolyzing O-glycosyl compounds (GO:0004553); carbohydrate metabolic process (GO:0005975); defense response (GO:0006952); cell wall organization (GO:0007047); embryo development ending in seed dormancy (GO:0009793)	-0.65	0.003182	0.181011
CR354742	PITA_000002063-RA	AT3G49720	259	4e-69	pectin methyl esterase CGR2	-	-0.65	3.87E-05	0.013359
CR392399	PITA_000023527-RA	AT5G15780	46	6e-05	Pollen Ole e 1 allergen and extensin family protein	-	-0.65	0.012003	0.329257
BX666019	PITA_000038835-RA	AT4G27670	39	6e-04	Heat shock protein 21	response to stress (GO:0006950); protein folding (GO:0006457)	-0.65	0.031262	0.488948
BX679064	PITA_000000268-RA	AT1G25510	461	1e-130	Eukaryotic aspartyl protease family protein	-	-0.65	0.013218	0.349164
AL749722	PITA_000031653-RA	AT2G39050	181	8e-46	hydroxyproline-rich glycoprotein family protein (EULS3)	-	-0.65	6.63E-05	0.017524
BX253632	PITA_000040483-RA	AT2G14900	94	9e-20	Gibberellin-regulated family protein GASA7	signal transduction (GO:0007165)	-0.64	0.00798	0.292513

BX254056	PITA_000000204-RA	AT2G03200	365	1e-101	Eukaryotic aspartyl protease family protein	biosynthetic process (GO:0009058); transport (GO:0006810); anatomical structure development (GO:0048856); cell wall organization or biogenesis (GO:0071554); carbohydrate metabolic process (GO:0005975); cellular protein modification process (GO:0006464)	-0.64	0.011411	0.326025
AL750591	PITA_000030264-RA	AT4G30270	274	6e-74	XYLOGLUCAN ENDOTRANSGLUCOSYLASE/HYDROLASE 24 (MERI-5)	signal transduction (GO:0007165); cell wall organization or biogenesis (GO:0071554); carbohydrate metabolic process (GO:0005975); aging (GO:0007568)	-0.63	0.039149	0.531339
BX678356	PITA_000031622-RA	AT1G59590	47	2e-05	ZCF37 (ZCF37)	-	-0.62	0.011435	0.326025
BX677658	PITA_000009345-RA	AT5G59310	67	1e-11	LTP4 | lipid transfer protein 4	response to stress (GO:0006950); transport (GO:0006810)	-0.62	0.000378	0.054605
BX250678	PITA_000007273-RA	AT2G37870	136	1e-32	Bifunctional inhibitor/lipid-transfer protein/seed storage 2S albumin superfamily protein	transport (GO:0006810)	-0.62	0.000364	0.054605
CR354677	PITA_000068716-RA	AT5G43150	34	0.000815	MMG4.18	-	-0.60	0.004721	0.218645
BX678418	PITA_000019323-RA	AT1G08480	99	2e-21	succinate dehydrogenase subunit (SDH6)	small molecule metabolic process (GO:0044281); generation of precursor metabolites and energy (GO:0006091)	-0.60	0.043167	0.29062
BX666046	PITA_000059169-RA	AT5G44550	118	4e-33	CASP-like protein 1B1	-	-0.60	0.027134	0.466653
BX680071	PITA_000010580-RA	AT5G67300	228	4e-60	myb-related protein MYB44	biosynthetic process (GO:0009058); response to stress (GO:0006950); cellular nitrogen compound metabolic process (GO:0034641); signal transduction (GO:0007165)	-0.60	0.008828	0.298543
CR393500	PITA_000023784-RA	AT3G53980	124	4e-29	Bifunctional inhibitor/lipid-transfer protein/seed storage 2S albumin superfamily protein	transport (GO:0006810)	-0.59	0.002531	0.165619

BX680617	PITA_000074822-RA	AT2G02148	90	3e-19	PPR containing protein	-	-0.59	0.007775	0.291848
BX253464	PITA_000046315-RA	AT1G29690	760	0	CAD1 MAC/Perforin domain-containing protein	plasma membrane (GO:0005886); defense response by callose deposition (GO:0052542); immune response (GO:0006955); cell death (GO:0008219); plant-type hypersensitive response (GO:0009626); response to salt stress (GO:0009651); response to salt stress (GO:0010337)	-0.59	0.03529	0.505357
BX677163	PITA_000015458-RA	AT1G60420	582	0	NUCLEOREDOXIN (NRX1)	integral component of membrane (GO:0016021); plasma membrane (GO:0005886); protein serine/threonine kinase activity (GO:0004674); signaling receptor activity (GO:0004872); protein binding (GO:0005515); ATP binding (GO:0005524); gametophyte development (GO:0048229); protein phosphorylation (GO:0006468); regulation of meristem structural organization (GO:0009934); regulation of meristem growth (GO:0010075); microsporocyte differentiation (GO:0010480)	-0.57	0.003922	0.199818
BX252568	PITA_000056860-RA	AT1G59620	32	0.72	disease resistance protein CW9	response to stress (GO:0006950)	-0.56	0.005022	0.226706
BX680131	PITA_000023780-RA	AT3G53980	129	1e-30	Bifunctional inhibitor/lipid-transfer protein/seed storage 2S albumin superfamily protein	transport (GO:0006810)	-0.56	0.001178	0.109134
BX250674	PITA_000015086-RA	AT2G37590	130	7e-35	f-box family protein [Populus trichocarpa]	nucleolus (GO:0005730); nucleus (GO:0005634); zinc ion binding (GO:0008270); DNA binding (GO:0003677); regulation of transcription, DNA-templated (GO:0006355); response to chitin (GO:0010200)	-0.55	0.006287	0.258398

BX681292	PITA_00000534 9-RA	AT1G11545	351	2e-121	XTH8 xyloglucan endotransglucosylase /hydrolase 8	apoplast (GO:0048046); cell wall (GO:0005618); xyloglucan:xyloglucosyl transferase activity (GO:0016762); hydrolase activity, hydrolyzing O-glycosyl compounds (GO:0004553); cellular glucan metabolic process (GO:0006073)	-0.56	0.003198	0.181011
BX679131	PITA_00002235 5-RA	AT4G22880	409	1e-114	leucoanthocyanidin dioxygenase (LDOX)	biosynthetic process (GO:0009058); response to stress (GO:0006950); secondary metabolic process (GO:0019748)	-0.55	0.020976	0.422111
BX253603	PITA_00003547 4-RA	AT3G29200	372	7e-129	CM3, cm-3 chorismate mutase 3	plastid (GO:0009536); cytosol (GO:0005829); chloroplast (GO:0009507); chorismate mutase activity (GO:0004106); response to biotic stimulus (GO:0009607); tryptophan biosynthetic process (GO:0000162); aromatic amino acid family biosynthetic process (GO:0009073)	-0.54	0.017955	0.400183
BX252727	PITA_00001127 5-RA	AT4G01130	219	6e-69	GDSL esterase/lipase	plant-type cell wall (GO:0009505); hydrolase activity, acting on ester bonds (GO:0016788); transferase activity (GO:0016740); lipid metabolic process (GO:0006629); defense response (GO:0006952)	-0.52	0.010367	0.315968
BX680496	PITA_00001429 7-RA	AT1G72740	77	1e-17	Telomere repeat-binding factor 5	nucleus (GO:0005634); nucleosome (GO:0000786); DNA binding (GO:0003677); nucleosome assembly (GO:0006334); regulation of transcription, DNA-templated (GO:0045449)	-0.52	7.21318E -06	0.004868
BX681305	PITA_00006003 5-RA	AT2G46600	118	6e-35	Calcium-binding EF-hand family protein	nucleus (GO:0005634); DNA binding (GO:0003677); calcium ion binding (GO:0005509)	-0.52	0.045365	0.559767
BX255011	PITA_00004645 4-RA	AT3G53980	113	8e-33	Bifunctional inhibitor/lipid-transfer protein/seed	peptidase activity (GO:0008233); lipid binding (GO:0008289); lipid transport (GO:0006869)	-0.52	6.84694E -06	0.004867 887

					storage 2S albumin superfamily protein				
CR393043	PITA_000059326-RA	AT5G60850	1267	1e-33	HCA2, DOF5.6 Dof-type zinc finger DNA-binding family protein	nucleus (GO:0005634); DNA-binding transcription factor activity (GO:0003700); protein binding (GO:0005515); zinc ion binding (GO:0008270); DNA binding (GO:0003677); regulation of transcription, DNA-templated (GO:0006355); positive regulation of transcription, DNA-templated (GO:0045941); response to cold (GO:0009409); response to light stimulus (GO:0009416); photomorphogenesis (GO:0009640); seed germination (GO:0009845); response to chitin (GO:0010200)	-0.51	0.047367	0.570158
BX250353	PITA_000083138-RA	AT4G29270	211	6e-55	HAD superfamily, subfamily IIIB acid phosphatase	biosynthetic process (GO:0009058); small molecule metabolic process (GO:0044281); lipid metabolic process (GO:0006629)	-0.51	6.6E-05	0.017524
BX680154	PITA_000073684-RA	AT2G41010	60	3e-10	Calmodulin-binding protein 25 (CAMBP25)	-	-0.51	0.017724	0.400183
BX252163	PITA_000022467-RA	AT2G39050	181	1e-45	hydroxyproline-rich glycoprotein family protein (EULS3)	-	-0.49	0.028769	0.476459
BX681579	PITA_000065922-RA	AT4G33510	817	0	DHS2 3-deoxy-d-arabino-heptulosonate 7-phosphate synthase	.	-0.49	0.009826	0.308708
CR394328	PITA_000042030-RA	AT2G20520	184	1e-57	FLA12 FASCICLIN-like arabinogalactan-protein 12	.	-0.49	0.000537	0.067235
BX254339	PITA_000009570-RA	AT3G26618	819	0	ARPN plantacyanin	apoplast (GO:0048046); obsolete plant extracellular matrix (GO:0048196); copper ion binding (GO:0005507); electron transfer activity (GO:0009055); anther development (GO:0048653);	-0.49	0.000369	0.054605

						transport (GO:0006810); pollination (GO:0009856); electron transport chain (GO:0022900)			
BX251279	PITA_000026545-RA	AT4G29100	155	5e-42	basic helix-loop-helix (bHLH) DNA-binding superfamily protein	-	-0.48	0.011110	0.322371
BX681956	PITA_000022625-RA	AT3G63410	203	4e-65	APG1, VTE3, IEP37, E37 S-adenosyl-L-methionine-dependent methyltransferases superfamily protein	-	-0.48	0.005091	0.228378
